# The functional head of the Cambrian radiodontan (stem-group Euarthropoda) *Amplectobelua symbrachiata*

**DOI:** 10.1186/s12862-017-1049-1

**Published:** 2017-08-30

**Authors:** Peiyun Cong, Allison C. Daley, Gregory D. Edgecombe, Xianguang Hou

**Affiliations:** 1 Yunnan Key Laboratory for Palaeobiology, Yunnan University, 2 Northern Cuihu Road, Kunming, 650091 China; 2 Department of Earth Sciences, The Natural History Museum, Cromwell Road, London, SW7 5BD UK; 3 Institute of Earth Sciences, University of Lausanne, CH-1015 Lausanne, Switzerland

**Keywords:** Radiodonta, *Amplectobelua*, Feeding structures, Functional head, Chengjiang

## Abstract

**Background:**

Segmental composition and homologies of the head of stem-group Euarthropoda have been the foci of recent studies on arthropod origins. An emerging hypothesis suggests that upper-stem group euarthropods possessed a three-segmented head/brain, including an ocular segment (protocerebrum) followed by the deutocerebrum with associated antennae/raptorial limbs and the tritocerebrum, while in the lower stem, head structures of Radiodonta are wholly associated with the protocerebrum and its preceding part. However, this hypothesis is incompletely tested because detailed knowledge on the head components of radiodontans is patchy, and informative articulated specimens are lacking for many taxa. *Amplectobelua symbrachiata* is the most common radiodontan species in the Chengjiang biota (ca. 520 Ma), normally known as isolated frontal appendages. Here we present detailed descriptions of new articulated specimens that elucidate the morphology and function of its head structures, and discuss their implications for hypotheses about euarthropod cephalic organisation.

**Results:**

In addition to a central oval head shield, *A. symbrachiata* also bears a pair of P-elements connected by an elongated rod. The mouth consists of sets of smooth and tuberculate plates, in contrast to the typical radial oral cones of other radiodontans. Previously identified ‘palm-like teeth’ are located external to the mouth in the posterior head region, and are interpreted as segmental gnathobase-like structures (GLSs) associated with at least three reduced transitional flaps in a one (pair)-to-one (pair) pattern, consistent with an appendicular nature. Comparisons with other panarthropods show that GLSs are morphologically similar to the mandibles and other gnathobasic mouthparts of euarthropods, as well as to the jaws of onychophorans, indicating their functional integration into the feeding activities of *A. symbrachiata*.

**Conclusions:**

The functional head of *A. symbrachiata* must include the reduced transitional segments (and their associated structures), which have been identified in several other radiodontans. This functional view supports the idea that the integration of segments (and associated appendages) into the head region, probably driven by feeding, occurred along the euarthropod stem-lineage. However, the number of reduced transitional segments varies between different groups and it remains uncertain whether GLSs represent proximal or distal parts of appendages. Our study is the first description of appendicular structures other than the frontal appendages in the functional head region of radiodontans, revealing novel feeding structures in the morphological transition from the lower- to the upper- stem-group of Euarthropoda.

## Background

Euarthropods are the most diverse and successful animal phylum ever to have lived on Earth. One of the key innovations that contributes to their evolutionary success is the integration of different appendages, together with the associated segments, into the head region, often to facilitate sensory (e.g. the antenna(e) of Mandibulata) and feeding activities (e.g. the chelicerae and pedipalps of most chelicerates, the maxillae and mandibles of Mandibulata, the forcipules of centipedes and maxillipeds of various crustaceans). However, the exact evolutionary history of these anatomical innovations remains obscure, causing an ‘endless dispute’ on the homology of head segments within and between euarthropods and their relatives, the onychophorans and tardigrades [1]. This dispute is difficult to settle because the morphologies of head segments and appendages are often highly modified or specialized, and as a result, there are no concordant criteria for recognising the head of Panarthropoda sensu Nielsen, 1995 [2], i.e., euarthropods, onychophorans and tardigrades [3, 4]. Functionally, the tagma of the panarthropod head region contains a mouth with associated structures for manipulation, tearing or mincing food, and usually some sensory organs (eyes, antennae and the associated ganglia, etc.) [3]. This functional view does not define the number of segments composing a head, which might cause confusion when comparing across panarthropods, but allows us to treat the panarthropod head tagma as a dynamic evolutionary unit that can be used to track how the different lineages of crown group euarthropods gained their head segments and associated structures step by step along their stem groups. For example, the integration of deutocerebral appendages (modified limbs) into the head is argued to be one of the key characters gained by the upper-stem euarthropods (Megacheira, Fuxianhuiida and Cambrian bivalved arthropods), and is regarded as a major evolutionary step towards crown-group euarthropods [5–7].

Comparison of head segments between crown-group euarthropods, onychophorans and tardigrades is now well anchored by the agreement that all these groups bear a protocerebral segment with associated eyes, although the presence/morphology of its attached limbs varies between different groups (see [8–10] for reviews). This homologous landmark provides a basis on which to homologize the head segments and associated structures of euarthropod stem group taxa with their various living relatives [7, 11–13]. In the nomenclature applied to the euarthropod stem following Ortega-Hernández 2016 [6]. Radiodonta is putatively a part of lower-stem group Euarthropoda (but see [14, 15] for an alternative view in which radiodontans are crown-group Euarthropoda) that bears one pair of arthropodized appendages in the head region, immediately in front of paired stalked eyes that correspond to the protocerebral segment. This pair of frontal appendages has been interpreted as being innervated by ganglia anterior to the protocerebrum, which is more comparable with the “antenna” of onychophorans [13, 16]. In addition, head carapaces of radiodontans, such as the head shield of *Anomalocaris canadensis* [17] and *Lyrarapax* [13, 18], as well as P- and H-elements of *Hurdia* [19, 20], were also inferred to be associated with the protocerebral segment [11]. Based on these observations, it has been suggested that the head of these radiodontans terminates functionally and anatomically behind the protocerebral segment [5].

The mouth, another key component of the functional head of radiodontans, is located on the ventral side of the head region and consists of a radial oral cone that has been identified in almost all taxa known from articulated specimens, including *Anomalocaris canadensis* [17, 21], *Hurdia victoria* [19, 20] and *Peytoia nathorsti* [22]. The radial oral cone has thus been considered as a key diagnostic character of Radiodonta [23], distinguishing it from the upper stem group and crown group of Euarthropoda (Deuteropoda sensu Ortega-Hernández 2016 [6]). However, a recently described radiodontan, *Lyrarapax* (with two known species, *L. unguispinus* and *L. trilobus*, both from the Chengjiang Konservat-Lagerstätte), shows a mouth without a radial oral cone, but rather composed of a series of concentric folds and furrows that form a sub-rectangular shape, contradicting the inference that a radial oral cone is a synapomorphy of radiodontans [13, 18]. Intriguingly, radially arranged oral lamellae or plates have also been identified in several lobopodians, such as *Hallucigenia* [24], *Jianshanopodia* [25] and *Pambdelurion* [26], indicating it might be a character with a deeper origin in Panarthropoda instead of being unique to Radiodonta. Nonetheless the morphology of the mouth of *Lyrarapax* indicates that some radiodontans resemble euarthropods and their upper stem group in lacking a radial oral cone, although the homology of such an absence cannot yet be determined. This raises several important questions concerning the early evolution of Euarthropoda, specifically questioning how the morphology of the mouth transformed from the lower stem group to the upper stem group, and whether there are other mouth types in Radiodonta.

*Amplectobelua symbrachiata* is a radiodontan described from nearly complete bodies from the Chengjiang biota [27, 28]. Like other radiodontans, the head of *A. symbrachiata* consists of a pair of frontal appendages [27], a pair of stalked eyes [28], and a head shield that was recognized only very recently [29]. However, its mouth has been described as composed of “large, elaborate teeth” ([28], p. 1306), or “palm-like jaws” ([30], p. 78; [﻿﻿31], p. 200) and smooth/tuberculated plates [28, 30, 31], which is different from the typical radial oral cones. In addition, the biological association of these structures with the body of *A. symbrachiata* has been questioned [27]. Even if such an association were verified, the homology of this mouth apparatus with the typical radiodontan oral cone needs to be appraised. Here we present a detailed description of the morphology of *A. symbrachiata*, with a focus on its head region, based on articulated specimens collected over the last two decades. We confirm the biological association of the ‘palm-like jaws’ and smooth/tuberculated plates with *A. symbrachiata*, re-interpret the ‘palm-like jaws’ as gnathobase-like structures (GLSs) of appendicular nature, and propose that smooth/tuberculated plates comprise the real mouth apparatus. Some additional head components, such as P-elements connected by a rod-shaped plate, are also described for the first time. Our study demonstrates that the functional head of *A. symbrachiata* not only has components shared with other radiodontans, such as frontal appendages, stalked eyes and head carapace(s), but also has three pairs of appendicular GLSs attached to the reduced transitional segments to facilitate tearing/mincing of prey.

## Results

### Systematic palaeontology

Total-group EUARTHROPODA Lankester, 1904 [32]

RADIODONTA Collins, 1996 [23]

AMPLECTOBELUIDAE Vinther, Stein, Longrich, Harper, 2014 [33]

*AMPLECTOBELUA* Hou, Bergström, Ahlberg, 1995 [27]

#### Type species

*Amplectobelua symbrachiata* Hou, Bergström, Ahlberg, 1995 [27]

#### Revised diagnosis of genus

Amplectobeluid with frontal appendage bearing pairs of spine-like endites generally devoid of auxiliary spines; one endite near proximal end stout and exceptionally long, one-third to nearly half as long as the length of the appendage; frontal carapaces include a central head shield and a pair of lateral P-elements connected by a rod-shaped plate; P-elements of similar size to head shield; mouth apparatus including smooth and tuberculate plates; three pairs of GLSs associated with reduced transitional flaps.

#### Remarks

Daley and Budd [34] slightly revised the diagnosis of this genus to include an additional species from the Burgess Shale, *Amplectobelua stephenensis*, and confined it to the morphology of the frontal appendages due to the lack of detailed description of other body parts. They remarked on the morphology of the body in detail*,* with a special note on the ‘large, elaborate teeth’ or ‘palm-like jaws’ described in early literature [28, 30]. New material and re-examination of the holotype reveal that the mouth of *A. symbrachiata* is not of the *Peytoia*-type, but is mainly composed of smooth and tuberculate sclerotized plates, while the ‘palm-like jaws’ are actually GLSs of an appendicular nature (see Description and Discussion below). Other characters, such as those of the trunk, need detailed description before being used in the diagnosis [34].

*Amplectobelua symbrachiata* Hou, Bergström, Ahlberg, 1995 [27]

(Figs. 1, 2, 3, 4, 5, 6, 7, 8, 9a and 10)

**Fig. 1:**
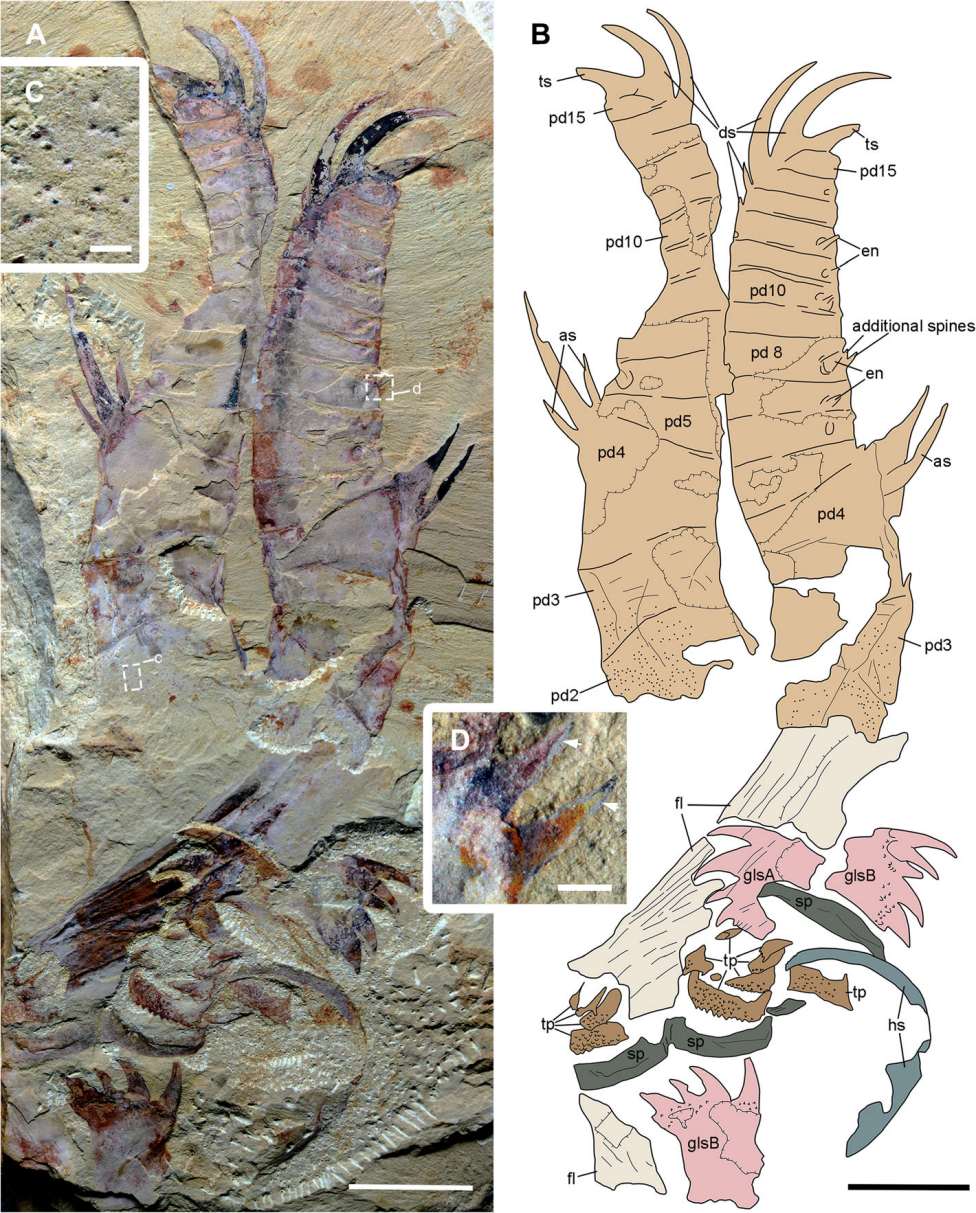
Functional head region of *Amplectobelua symbrachiata*. **a**, YKLP 13889, showing paired frontal appendages, gnathobase-like structures and mouth plates preserved together. **b**, interpretative drawing. **c**, close-up of small tubercles (boxed in a) on podomeres 2 and 3. **d**, close-up of small spines branched from the inner (ventral) medial-edge of podomere 8 (boxed in a). See Methods for abbreviations. Scale bars: a, b, 1 cm; c, d, 0.5 mm

**Fig. 2:**
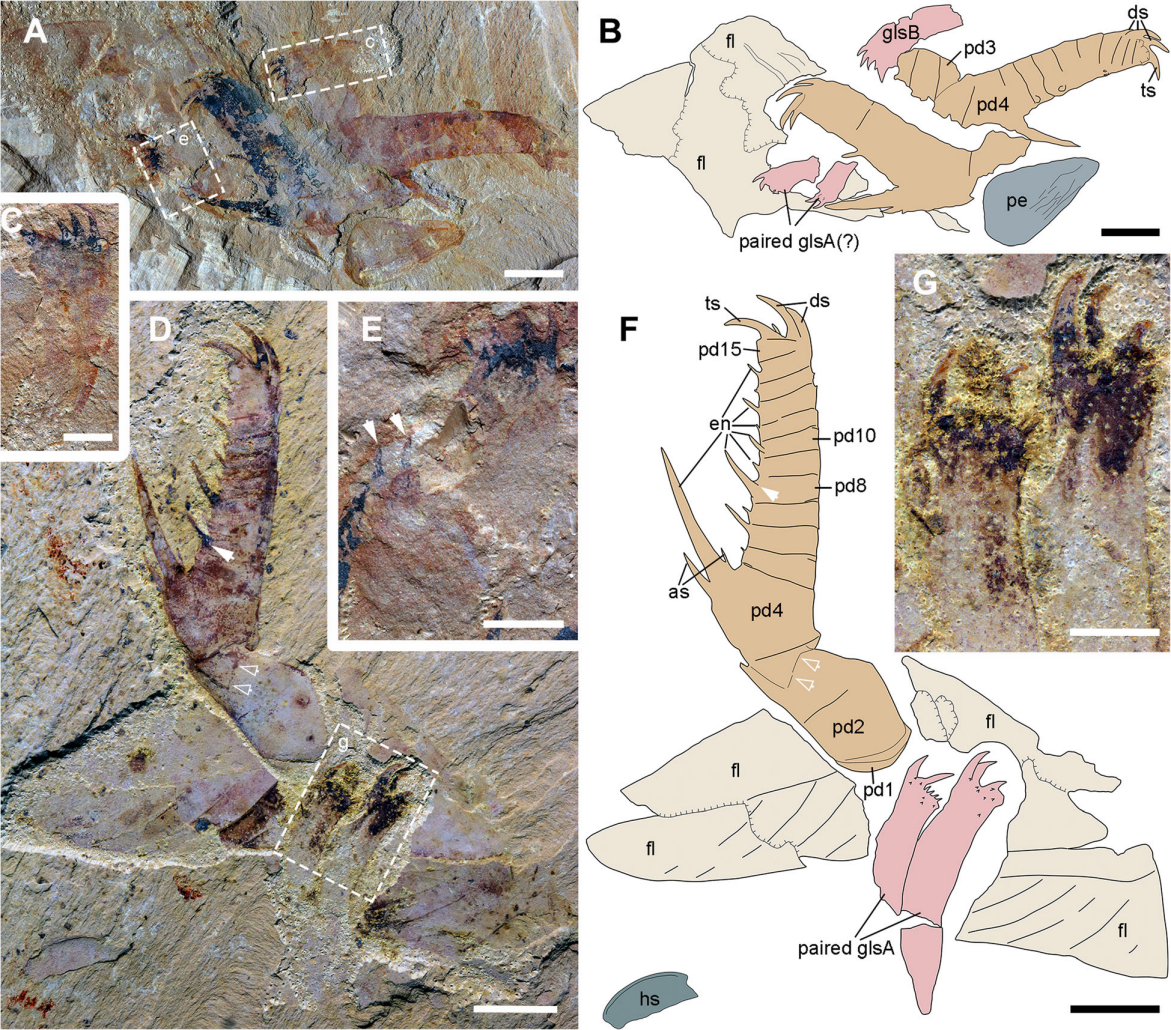
Gnathobase-like structures of *A. symbrachiata* preserved with the frontal appendages. **a**-**b**, YKLP 13314 and its interpretative drawing, showing a pair of GLSs and an isolated one, note the triangular sclerite (P-element) in the lower right corner. **c**, **e**, close-up of GLSs (boxed in a), arrows in e indicating two poorly preserved distal spines of a paired GLS. **d**, **f**, YKLP 13313a and its interpretative drawing, showing a pair of GLSs with a long stem, hollow arrows indicating possible inter-podomere membrane, solid arrow in d indicating the rising position of the endite. **g**, detail of the paired GLSs (boxed in d). See Methods for abbreviations. Scale bars: a, b, d, f, 5 mm; c, e, g, 2 mm

**Fig. 3:**
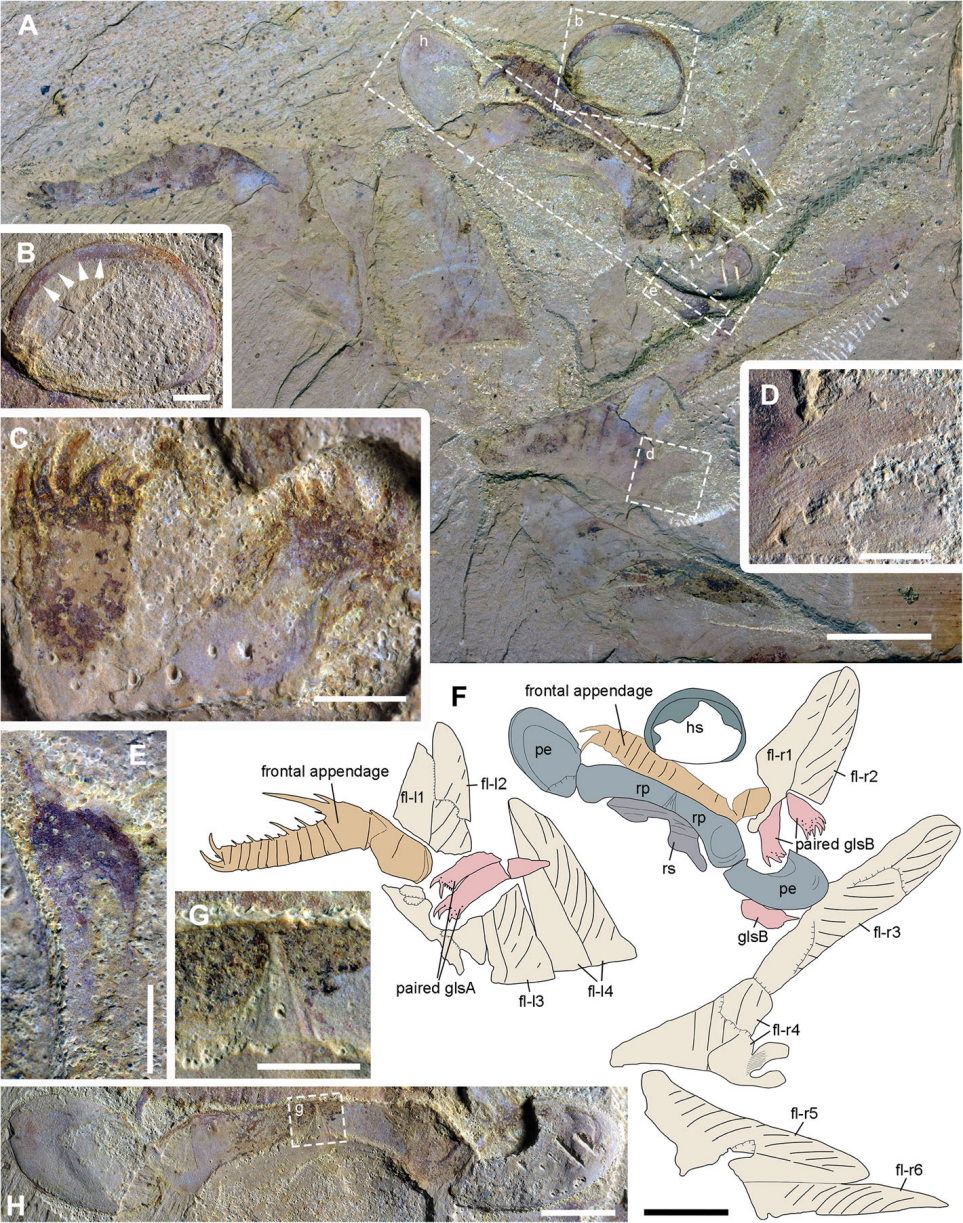
Head carapace, gnathobase-like structures and body flaps of *A. symbrachiata*. **a**, YKLP 13313b. **b**, close-up of the head shield (boxed in a), arrows indicating the rim of the head shield. **c**, detail of another pair of GLSs (boxed in a). **d**, close-up of the basal extensions of a flap (boxed in a), note the filaments on the surface. **e**, close-up of an isolated GLS (boxed in a). **f**, combined interpretative drawing of YKLP 13313a, b. **g**, close-up of the triangular connecting region in the middle of the dumbbell shaped sclerite (boxed in h). **h﻿**, close-up of the pair of P-elements and the intermediate rod-shaped sclerites. See Methods for abbreviations. Scale bars: a, f, 1 cm; b-e, g, 2 mm; h, 5 mm

**Fig. 4:**
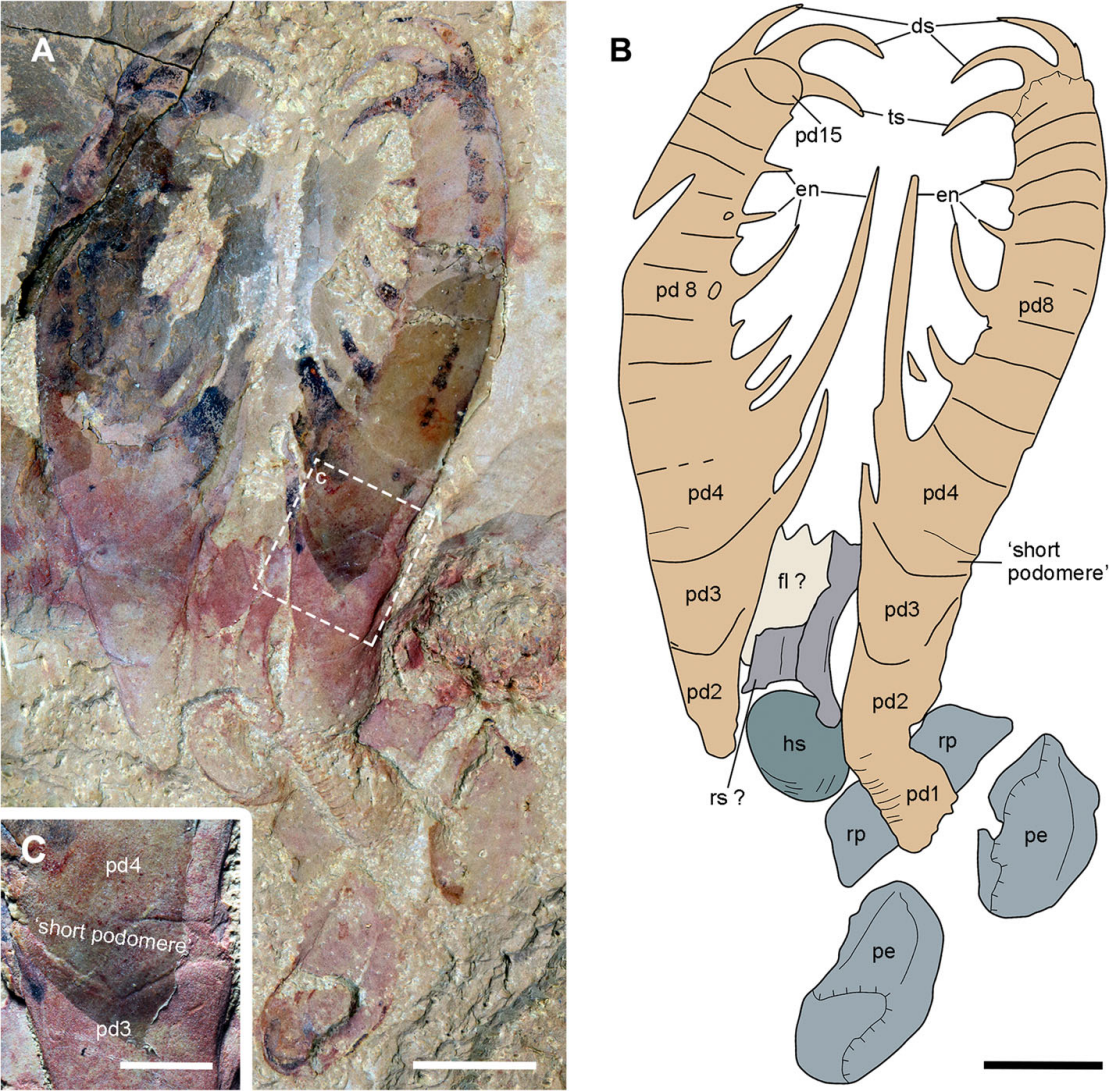
Holotype of *A. symbrachiata*. **a**, NIGPAS 115346. **b**, interpretative drawing. **c**, close-up of the ‘small podomere’ between podomeres 3 and 4. See Methods for abbreviations. Scale bars: a, b, 5 mm; c, 2 mm

**Fig. 5:**
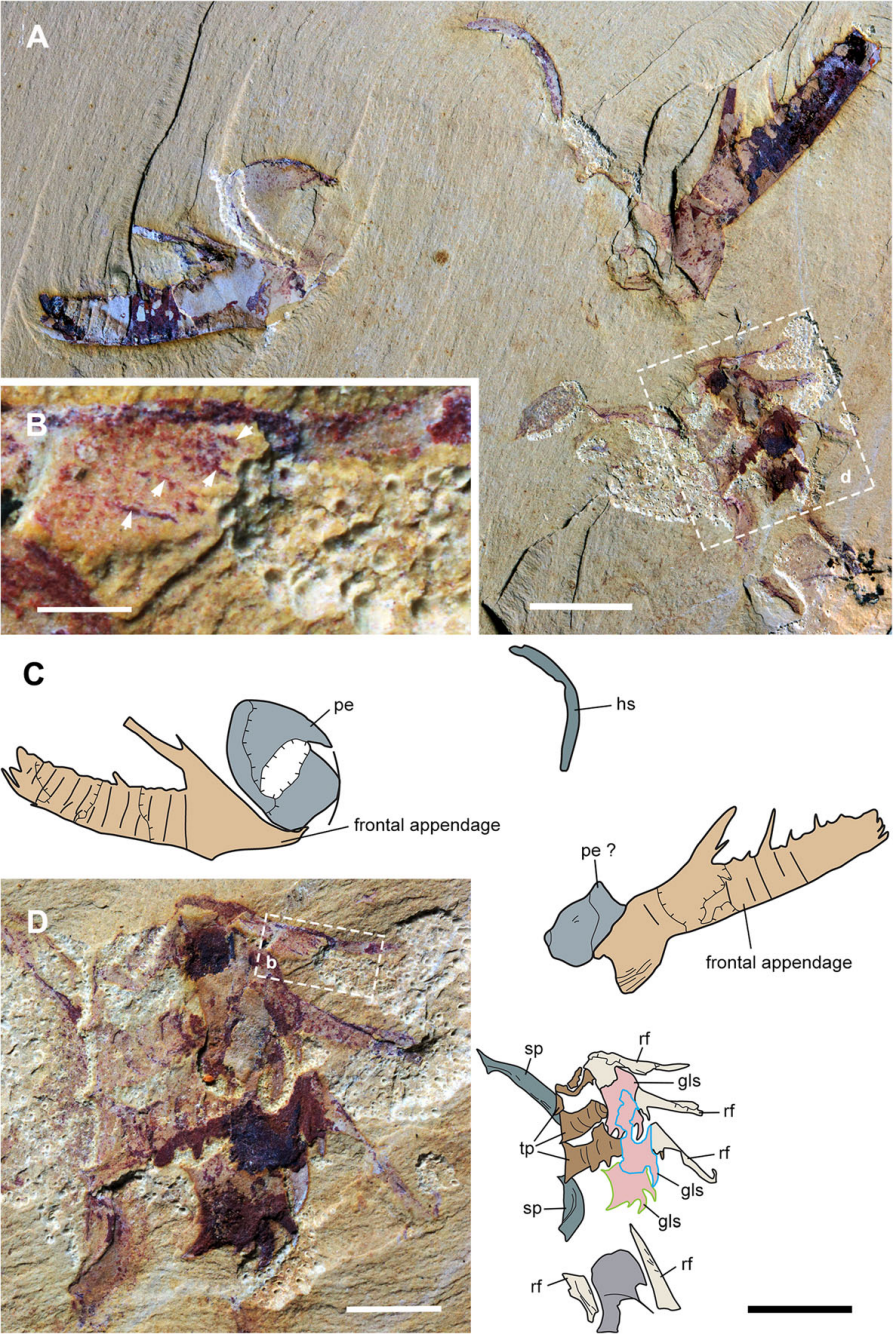
Alignment of gnathobase-like structures and reduced transitional flaps of *A. symbrachiata*. **a**, YKLP 13323a. **b**, close-up of a reduced transitional flap (boxed in d), arrows indicating the transverse lines. **c**, interpretative drawing of a. **d**, close-up of reduced transitional region (boxed in a). See Methods for abbreviations. Scale bars: a, c, 5 mm; b, 0.5 mm; c, 2 mm

**Fig. 6:**
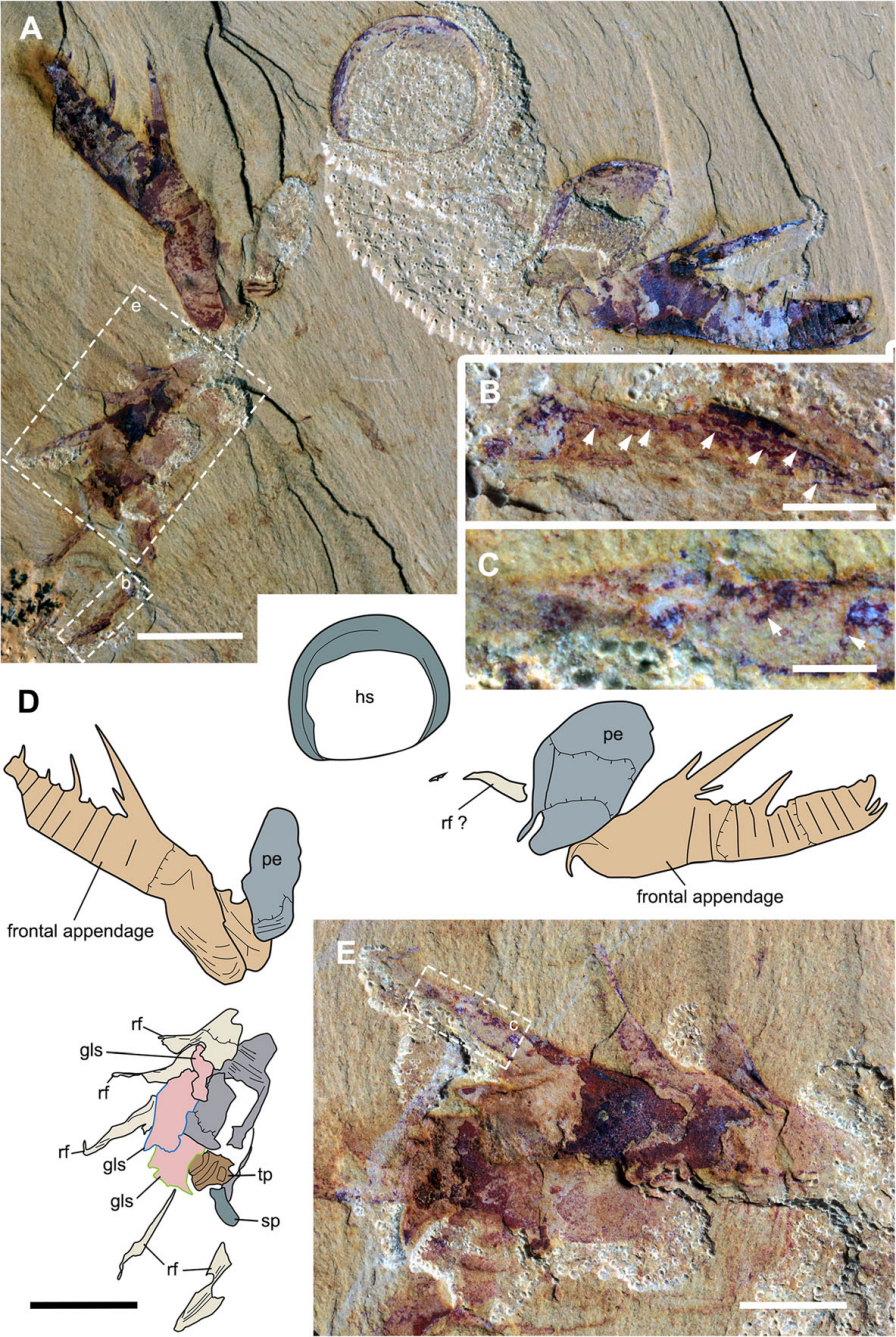
*A. symbrachiata*, counterpart of YKLP 13323. **a**, YKLP 13323b. **b**-**c**, close-up of a reduced transitional flap (boxed in a and e, respectively), arrows indicating the transverse lines. **d**, interpretative drawing of a. **e**, close-up of reduced transitional region (boxed in a). See Methods for abbreviations. Scale bars: a, d, 5 mm; b, c, 0.5 mm; e, 2 mm

**Fig. 7:**
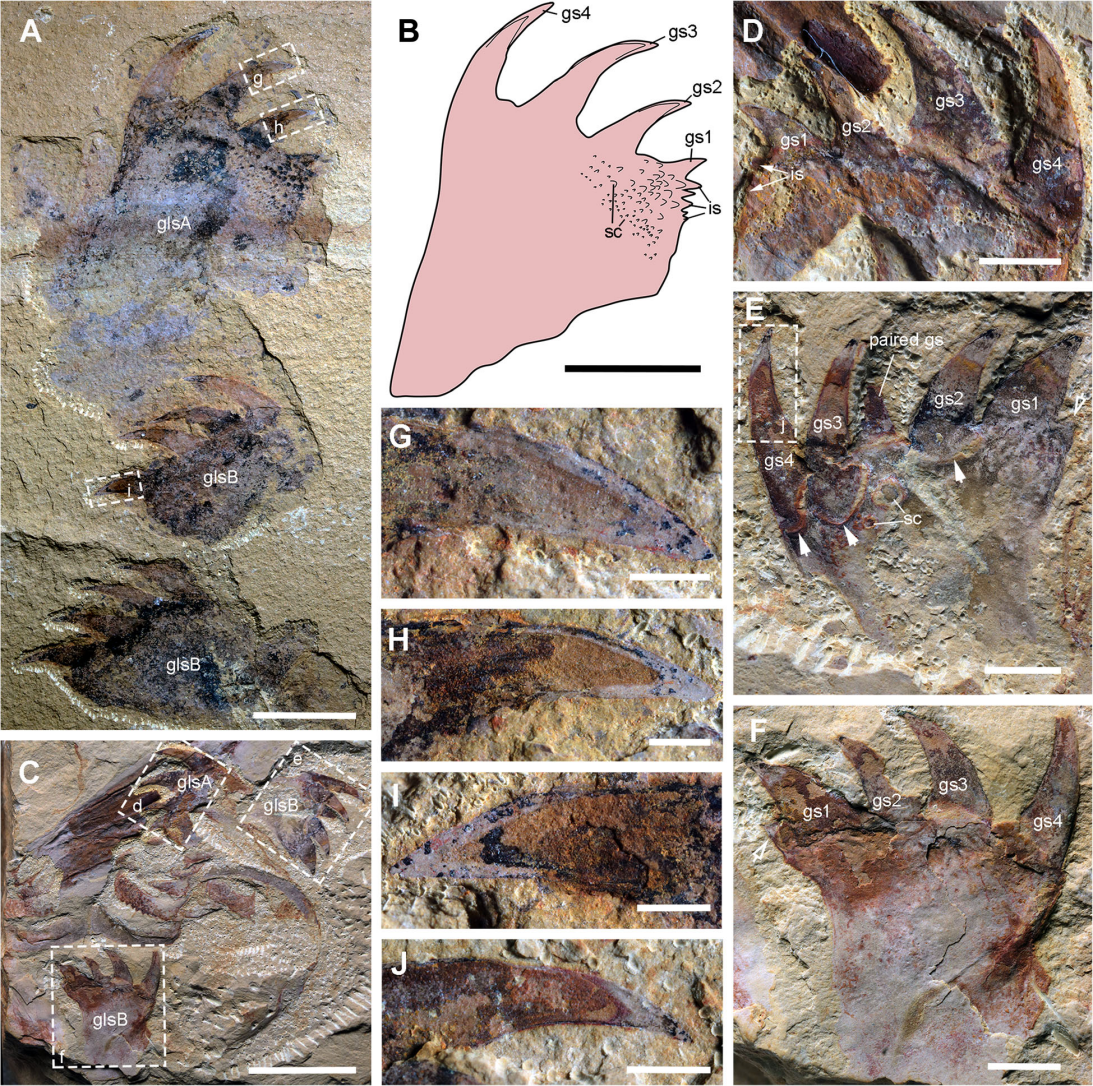
Morphology of the gnathobase-like structures of *A. symbrachiata*. **a**, YKLP 13317, showing three isolated GLSs preserved in a row. **b**, interpretative drawing of the top GLS (morph-A, see main text) in a. **c**, close-up of the mouth region of YKLP 13889. **d**, close-up of the morph-A GLS of YKLP 13889 (boxed in c). **e-f**, close-up of the morph-B GLSs of YKLP 13889 (boxed in c), hollow arrows showing the sharp meeting point of the inner and distal edges, note that distal spines are set within sockets as indicated by the linear swollen region (solid arrows). **g**-**j**, close-up of distal spines showing cone-in-cone pattern, g-i, from YKLP 13313, boxed in a; j, from YKLP 13889, boxed in e. See Methods for abbreviations. Scale bars: a-c, 1 cm; d-f, 2 mm; g-j, 1 mm

**Fig. 8:**
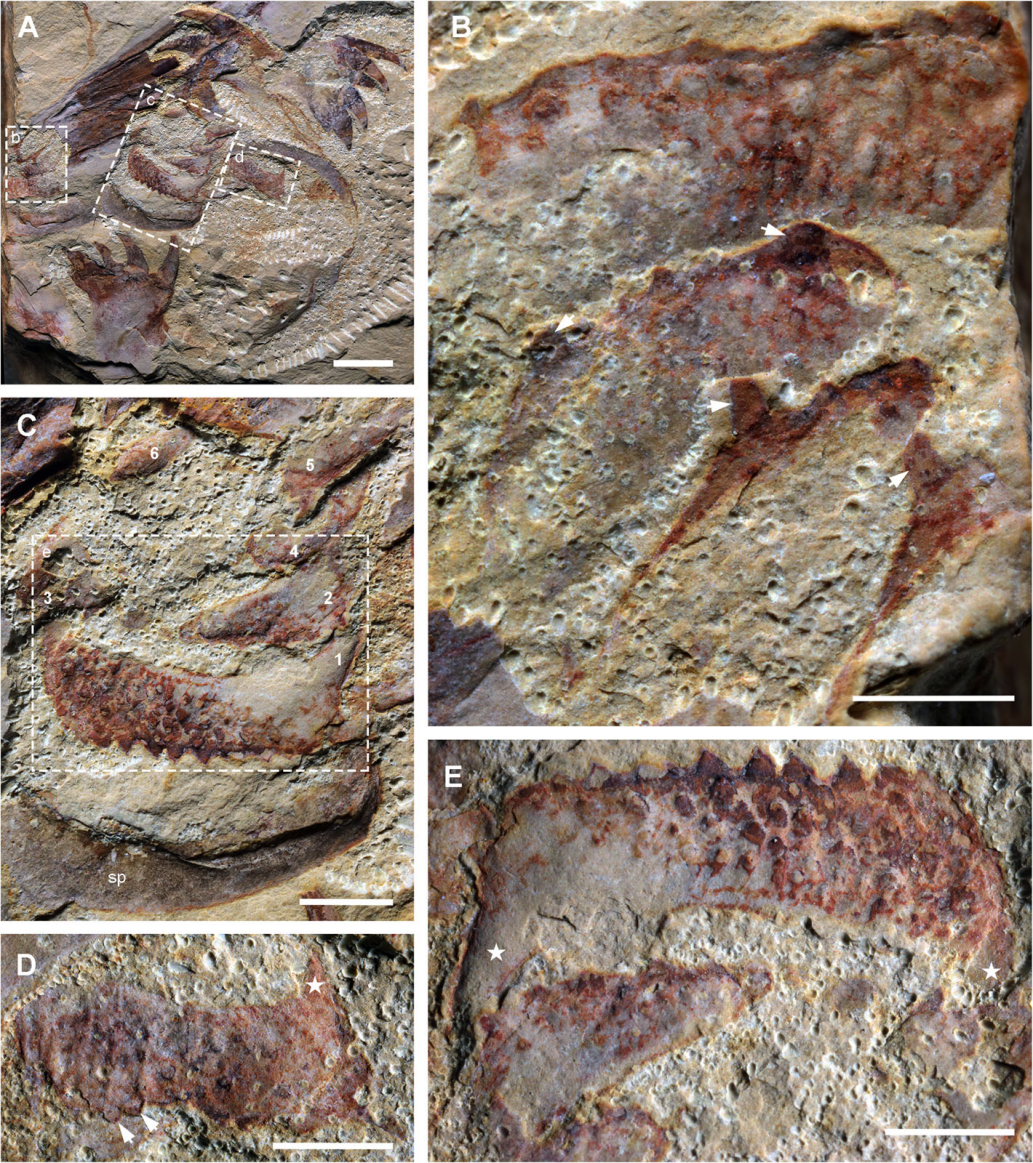
Mouth plates of *A. symbrachiata*. **a**, close-up of the mouth region of YKLP 13889. **b**, close-up of the left row of tuberculate plates (boxed in a, orientation rotated by 180°), arrows indicating the large spines along the outer edge of the plates. **c**, close-up of the middle row of tuberculate plates (boxed in a) numbered 1–6. **d**, close-up of the right row of tuberculate plates, note the posteriorly pointed large spine at the posterior corner (star), arrows indicating spines along the outer edge. **e**, close-up of the rectangular tuberculate plate (boxed in c, rotated 180°), star showing the backwards pointed large spine at the posterior corner. See Methods for abbreviations. Scale bars: a, 5 mm; b-e, 2 mm

**Fig. 9:**
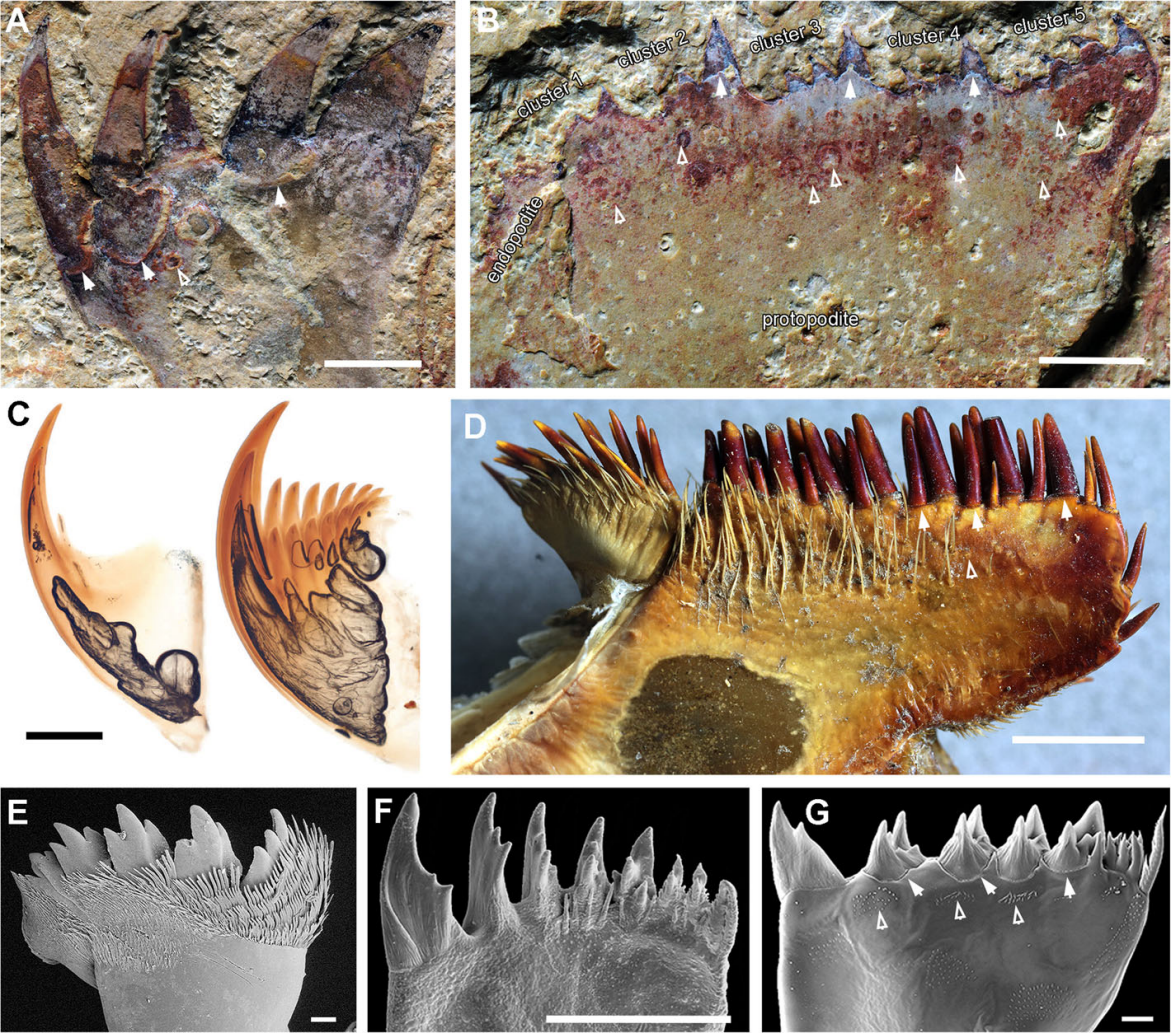
Comparison of GLSs and similar feeding structures of selected panarthopods. **a**, GLS of *A. symbrachiata*, solid arrows indicating swollen sockets, hollow arrows indicating scales. **b**, gnathobase of *Parapeytoia yunnanensis*, showing 5 clusters of spines along the gnathal edge of protopodite, solid arrows indicating double layers at the base of distal spines, hollow arrows indicating scales along the gnathal edge. **c**, inner (right) and outer (left) blades of the jaw of extant onychophoran, *Euperipatoides kanangrensis*, images courtesy of Martin Smith and Javier Ortega-Hernández, note the stacked constituent elements. **d**, gnathobase of the right third walking limb of the xiphosuran *Limulus polyphemus*, viewed from the posterior, solid arrows indicating double layers at the base of distal spines, hollow arrows indicating scales along the gnathal edge. **e**, mandible of extant scolopendromorph centipede, *Ethmostigmus rubripes*. **f**-**g**, mandible of extant crustaceans (Copepoda), *Microcalanus pygmaeus* (f) and *Rhincalanus gigas* (g), images courtesy of Jan Michels, note the morphological differences between them due to the difference of diet [50], solid arrows indicating double layers at the base of distal spines, hollow arrows indicating scales along the gnathal edge. See Methods for abbreviations. Scale bars: a-b, 2 mm; c, e, 100 μm; d, 5 mm; f, g, 20 μm

**Fig. 10:**
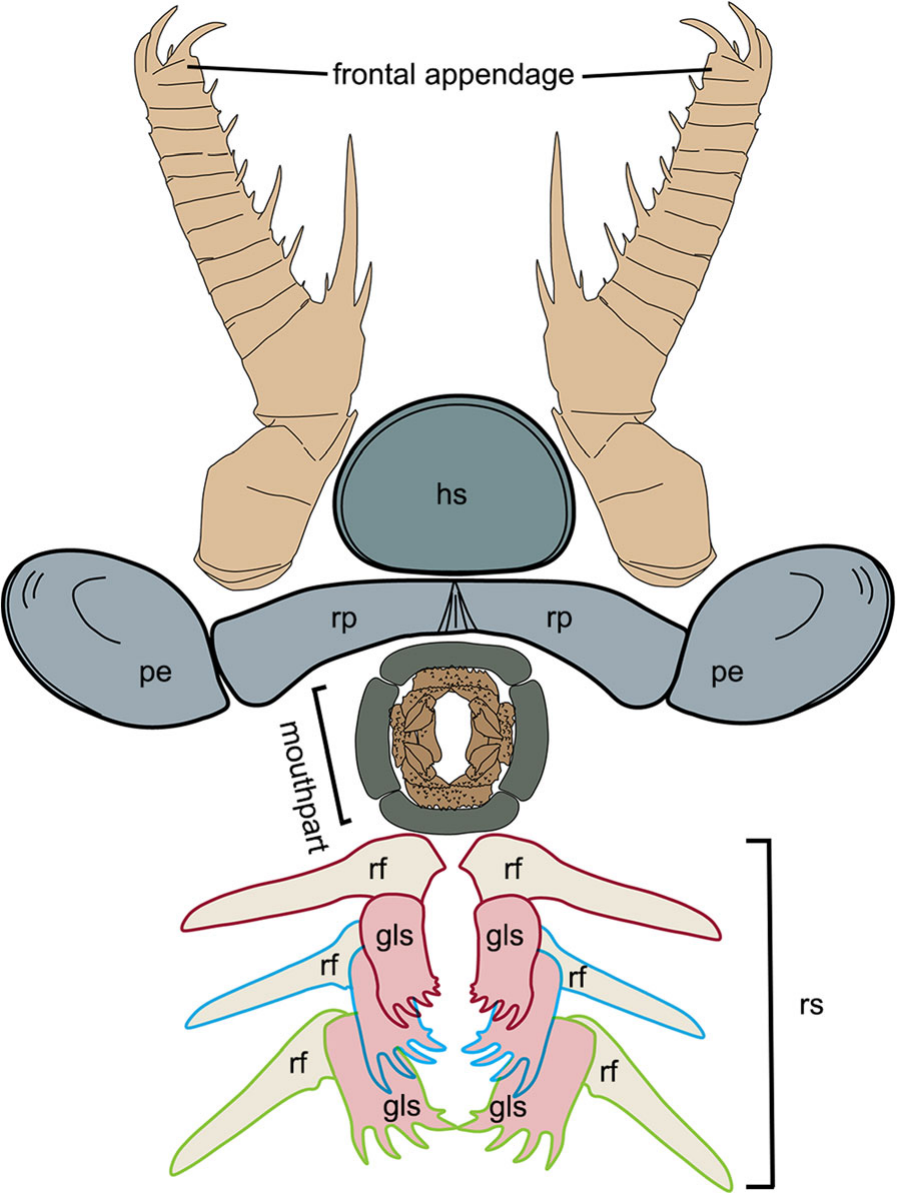
Reconstruction of the topological arrangement in the functional head region of *A. symbrachiata*. The reconstruction of the mouth follows that of other radiodontans, such as *Hurdia* and *Peytoia*, although the symmetry and the arrangement of mouth plates are conjectural. The relative position of the GLSs is based on their association with the reduced transitional flaps and inferred function (by comparison with gnathobases of euarthropods, see the main text). The correspondence between GLSs and the reduced transitional flaps is indicated by the coloured outline. The relative size of each component is based on specimens illustrated herein. The size of animal is not scaled. See Methods for abbreviations

1994 New anomalocaridid animal 2 from Chengjiang; Chen et al., p. 1306, fig. 3. [28]

v^*^ 1995 *Amplectobelua symbrachiata*; Hou et al., pp. 176–177, figs 14–15. [27]

1996 *Amplectobulua symbrachiaciata*; Chen et al., pp. 199–200, figs 267–272. [31]

1997 *Amplectobelus sumbrachiata*; Chen, Zhou, pp. 79–80, figs 125–128. [30]

v. 1999 *Amplectobelua symbrachiata*; Hou et al., p. 68, figs 83–84. [35]

1999 *Amplectobelua symbrachiata*; Luo et al., pl. 16, figs 2–6. [36]

1999 *Hipopotrum spinatus* Luo, Hu *in* Luo et al.; Luo et al., pl. 27, fig. 7. [36]

2002 *Amplectobelua symbrachiata*; Chen et al., pl. 14, figs 3–4. [37]

v. 2004 *Amplectobelua symbrachiata*; Hou et al., p. 97, fig. 15.3. [38]

2004 *Amplectobelua symbrachiata*; Chen, pp. 302–304, figs 483, 485–487. [39]

v. 2014 *Amplectobelua symbrachiata*; Cong et al., extended data fig. 1b-d. [13]

v. 2017 *Amplectobelua symbrachiata*; Hou et al., p. 157, fig. 19.3. [40]

2017 *Amplectobelua symbrachiata*; Zeng et al., p. 23, fig. 18e, f. [29]

? 2017 undetermined taxon (NIGPAS 162524); Zeng et al., p. 16, fig. 12. [29]

#### Holotype

NIGPAS 115346, the holotype in the original description, is a juvenile specimen (based on the relatively small size of the frontal appendage) with nearly complete frontal appendages, a head shield and P-elements. The morphology of frontal appendages is the same as all known adult specimens, being 2.5 cm from the distal vertex to the base of podomere 4 (see Description below).

#### Other new referred material

YKLP 13313, YKLP 13314, YKLP 13317, YKLP 13323 and YKLP 13889, five newly collected specimens that preserve frontal appendages and GLSs except YKLP 13317, in which only three isolated GLSs are preserved. Its assignment to this taxon is based on morphological similarities between the GLSs and those of other material (see Description below). All specimens are partially articulated and the outline of the animal is distorted. Their size is determined based on the size of the frontal appendage. In YKLP 13313, 13314, 13323 and 13889, the frontal appendage (from the distal vertex to the base of podomere 4) is about 2 cm, 1.8 cm, 1.2 cm and 4.3 cm, respectively. The width of GLSs in YKLP 13317 can reach ca. 1.8 cm, which is double the size of those in YKLP 13889. This indicates that the length of the frontal appendage of YKLP 13317 can reach at least 8.6 cm.

#### Locality and horizon

All specimens described herein were collected from the lower-middle part of the Yu’anshan Member, Chiungchussu Formation, Cambrian Series 2, Stage 3, in eastern Yunnan Province, China. The holotype NIGPAS 115346 is from the Maotianshan section, Chengjiang; YKLP 13313 is from the Ercaicun section, while YKLP 13314, YKLP 13317 and YKLP 13889 are from the Mafang section, both of which are located in Haikou, Kunming, and are adjacent to each other (see [40] for detailed stratigraphy and localities).

#### Revised diagnosis of species

*Amplectobelua* with frontal appendages bearing 15 podomeres; podomeres 3–15 having simple spine-like endites except on podomere 4, which has an extremely elongated endite that bears a pair of auxiliary spines branching from its most basal part; size of endites alternating, decreasing distally along both odd and even podomeres except on podomere 8; an additional large spine is present on the tip of podomere 15; dorsal spines present on last four distal podomeres, with the last two significantly larger; podomere 1 annulated and flexible; head shield oval, with posterior edge nearly straight; P-elements ovoid, with rod-shaped sclerite between them divided by a triangular region in the middle; mouth composed of radial rows of outer rectangular smooth plates and inner tuberculate plates; three pairs of GLSs, with small scale-like nodes on the blade, and four (pairs) of strong spines along the distal edge; trunk flaps with transverse lines confined to their anterior part.

#### Remarks

*Amplectobelua symbrachiata* differs from the only other congener, *A. stephenensis* from the Burgess Shale, in the number of podomeres on the frontal appendage (15 in *A. symbrachiata* versus 12 in *A. stephenensis*) and the endite morphology, especially the extremely large endite [34]. It is worth noting that the podomere that bears the largest endite in the two known species is different, i.e., podomere 4 in *A. symbrachiata* versus the most proximal podomere in *A. stephenensis*. This difference can either be caused by preservation or is of taxonomic value [34]. All characters observed in other body parts are here taken into the diagnosis of *A. symbrachiata*, although some of them might be shared characters with the other species. These characters can only be re-appraised when the body of *A. stephenensis* is found and described.

An enigmatic Chengjiang fossil, *Hipopotrum spinatus* Luo, Hu, 1999, was described very briefly based on one specimen collected from the Ercaicun section in the Haikou area [36]. It is only 1.5 cm wide, 3 mm long, with a horseshoe shape and decorated with small scales or tubercles on the surface. The supposed anterior edge bears 12 triangular spines while the posterior is smooth, with a distinct lateral spine at both corners. This morphology of *H. spinatus* is strikingly similar to one kind of tuberculate plate described here as part of the mouth region of *A. symbrachiata* in shape, size and ornamentation (see Description below). It is thus inferred that *H. spinatus* is an isolated mouth plate of *A. symbrachiata*, and accordingly the genus and species are considered junior synonyms of the latter.

### Description

#### Frontal appendages

Isolated frontal appendages of *Amplectobelua symbrachiata* are quite common in Chengjiang. They consist of 15 podomeres, as stated in the original description [27], and generally curve towards the ventral (inner) side that bears endites. The configuration of podomeres, especially in the proximal part, is slightly modified here. All podomeres are (sub) rectangular in shape except for podomere 4, where the ventral (inner) side with endites is nearly twice as long as the dorsal (outer) side; podomeres 1–4 are longer than podomeres 5–15 (Figs. 1a–b; 2d, f; 3a, f; 4a–b; 5a, c; 6a, d), normally bending outwards at an angle of around 100°, such that podomeres 1–3 form a ‘shaft’ of the appendage (Fig. 2d, f). Similar bending can also be observed between podomeres 1 and 2 (Fig. 4a–b). In most cases, podomere 1 is not visible, but when preserved it bears a series of parallel arcuate lines (Figs. 2d, f; 4a–b; 6a, d), which were previously interpreted as *Peytoia*-type mouth sclerites (striated structure) [27]. Examination of the holotype and new specimens shows that the surface and the outline of the ‘striated structure’ is continuous with adjacent succeeding podomeres (Figs. 2d, f; 4a–b). In addition, the real mouth of *A. symbrachiata* is not a *Peytoia*-type (see Description and Discussion below). These lines of evidence together indicate that a podomere, probably annulated as indicated by the parallel arcuate lines, is present at the most basal part of the frontal appendage. An additional ‘short podomere (segment)’ preceding podomere 4 previously described in the holotype is questionable (Fig. 4a–c), as it cannot be confidently identified in other known specimens. However, podomere 4 in the holotype does not bend at an angle to adjacent ones, an unusual posture that must have altered their shape as indicated by the curved posterior boundary of podomere 3 and the unusually narrower proximal part of the frontal appendage (Fig. 4a–c). Thus the ‘short podomere’ is likely a result of taphonomic artefacts. This interpretation is supported by the position of the supposed boundary between it and podomere 4, which is located right at the base of the spine (not the proximal auxiliary spine) of podomere 4 (figs 14a–b, 15 a-b in [27]). This is, however, not the case in other known specimens, where it is located far from the endite spines (Fig. 2d, f). Interestingly, there is a linear structure nearly transversely crossing the distal part of podomere 3 in YKLP 13313, forming a podomere-like shape (hollow arrows in Fig. 2d, f). This structure is regarded as similar to, but not same as, the ‘short podomere’ because the shape of podomere 3 indicates that it is located lower than that of the holotype specimen. An alternative interpretation for the ‘short podomere’ is that it is a soft arthrodial membrane required to facilitate the significant flexibility of podomere 4, which is suggested by its nearly vertical position relative to the shaft podomeres and its large size.

In specimen YKLP 13889, podomeres 2 and 3 are ornamented with many small tubercles (Fig. 1a–c). Simple endites are present on podomeres 3–15, all of which are shaped like a triangular spine except for that of podomere 4, which has a pair of auxiliary spines at the base (as in Figs. 1a–b; 2d, f). The endite on podomere 8 also has an additional pair of small spines, which do not branch from the main one (Fig. 1a–b, d). The endites originate from the ventral (inner)-medial region of the podomeres (solid arrows in Fig. 2d, f). The size of the endites alternates on successive podomeres, with those on the even podomeres being larger than those on the odd. Endites are sometimes broken off, leaving only round structures with relief along the ventral (inner) margin (Fig. 1a–b). In general, the size of endites on both odd and even podomeres decreases distally, except for podomere 8, which is slightly larger than the endite of podomere 6 (Fig. 2d, f). Each of podomeres 12–15 bears a dorsal (outer) spine, with those of podomeres 14–15 much larger and curving forward over the end of the appendage (ds in Fig. 1a–b). In addition, podomere 15 has one more large apical spine at the distal end (ts in Figs. 1a–b; 2d, f), which together with the dorsal spines of podomeres 14–15 form a claw-like end. The articulation and flexibility of the podomeres are the same as in early descriptions [27].

#### Gnathobase-like structures

“Large, elaborate teeth” ([28], p. 1306), or “palm-like jaws” ([30], p. 78; [﻿﻿31], p. 200) have been described in association with the frontal appendages of *Amplectobelua symbrachiata*, and have been mentioned as possible gnathobases [41]. Although the biological association has been doubted [27], additional specimens illustrated here (Figs. 1, 2, 3, 5 and 6) indicate that this is not a chance occurrence of unrelated material, and that they are parts of the same animal. These structures are described here as GLSs of an appendicular nature (see Discussion below).

The GLS is elongated in shape, with one end normally bearing four stout spines, here termed as distal. The width of GLSs is normally two-thirds the width of podomere 5 of the frontal appendages. Their length varies between and within individuals (partially due to preservation), sometimes reaching over three times the width (Fig. 3a, f). The four distal spines are aligned in a row and slightly curved, pointing to the same side, here termed inner (Figs. 1a–b; 2; 3a, c, e, f; 7a–f). In some specimens, this row overlies additional stout spines, indicating that one or more such spines are paired (Figs. 2g; 3c; 7e). Some distal spines show a cone-in-cone layering, the two layers sharply distinguished from each other mainly in coloration, with a lighter coloured outer layer that tapers out distally to the apex of the spine (Fig. 7g–j). Swellings can occasionally be recognized at the base of the distal spines, defined by distinct curved linear structures, indicating that the distal spines are set within sockets along the distal edge of the GLS (arrows in Figs. 7e; 9a). The length of the four distal spines decreases from the outer side to the inner side, with the length spectrum varying in different GLSs, which can accordingly divide GLSs into two types. In some GLSs, the length of the first outer spine reaches about half the GLS width, with that of the most inner spine reaching only one tenth of the GLS width. In these cases, the width of the spines also decreases in the same pattern as the length. Additional small spines can be observed along the inner side of this type of GLS, which is termed morph-A (glsA in Figs. 1a–b; 7a–d). In the other morphotype (morph-B), distal spine length does not decrease significantly, and the width increases from the outer side to the inner side, making the spine at the innermost side the stoutest (glsB in Figs. 1a–b; 7a, c, e, f).

The stem of the GLS is cylindrical and curves slightly to the inner side (Figs. 2; 3a, c, e, f). The outer and inner edges of the stem are nearly parallel, except in the distal region. In morph-A, the inner edge of the distal region is nearly straight and bears several inner spines with lengths that decrease proximally (is in Fig. 7b), and the outer edge expands laterally slightly to form a smooth curved edge continuing to the outermost distal spines (Fig. 7a, b). In morph-B, the outer edge of the distal region is similar to that of morph-A, while the inner edge expands to the inner side, making the distal region significantly wider than the proximal part of the stem. The meeting point of the inner and distal edges forms a sharp spine-like angle (hollow arrows in Fig. 7e, f). In morph-A, numerous scales/spines are present along the distal region of the GLS stem, and the sizes of these scales decrease proximally (sc in Fig. 7a–b). In morph-B, scales are also present but quite rare (sc in Fig. 7e).

In YKLP 13313, a total of five GLSs can be recognized from both part and counterpart slabs, four of which are arranged as adjacent pairs (Figs. 2d, f; 3a, f). In the part, a pair of morph-A GLSs is preserved adjacent to the base of a frontal appendage, arranged in the same orientation with their distal spines pointing in the same direction (Figs. 2d, f, g; 3f). In the counterpart, a second pair of morph-B GLSs is located at the base of another frontal appendage, arranged with their distal spines pointing in opposite directions (Fig. 3a, c, f). The contrasting orientation of these two GLS pairs indicates that they can rotate to some extent, and might be flexible when manipulating food. The unpaired GLS has only one large innermost spine preserved, indicating it is morph-B (Fig. 3a, e). Interestingly, this unpaired GLS is adjacent to a high-relief oval carapace (see Description below) and is located on a lower sediment lamina (Fig. 3a, f). Given the paired nature of other GLSs, it is reasonable to postulate that this GLS is also paired, with the opposite one being covered by the carapace. In YKLP 13889, three GLSs are preserved, with the top left one being of morph-A, the other two being morph-B (glsA/B in Fig. 1a–b). In YKLP 13314, there are also three GLSs preserved. The bottom two are probably morph-A and are arranged in a pair with the distal spines pointing opposite to each other (Fig. 2a–b, e). The top one with a long stem part is unequivocally morph-B, as evidenced by a stout innermost spine (Fig. 2a–c). The number of GLSs in *Amplectobelua symbrachiata* has been described as ‘at least six, perhaps eight, possibly set in pairs’ [25]. Based on the observation above, it is confirmed that this animal has at least three pairs of GLSs.

In YKLP 13323, there are three GLSs aligned anteroposteriorly in one row (Figs. 5a, c, d; 6a, d, e), with the distal spines pointing in same direction. The GLS row overlaps a region bearing three reduced transitional flaps that are also aligned in a row and in a consistent orientation (see Description below). The GLSs successively overlap each other, with the most posterior one on top. The most proximal parts of these three GLSs are well aligned with each of the three reduced transitional flaps, indicating that each GLS corresponds with one (pair of) flap(s). These lines of evidence suggest that the three GLSs are not paired partners to each other, but are instead from the same side of the animal. At the opposite side of the specimen, there is a series of plates that are interpreted as smooth and tuberculate plates from the mouth region (see Description below).

#### Tuberculate and smooth plates

In the earliest description of *Amplectobelua symbrachiata*, a “plate circle” ([28], p. 1306) was described preserved together with GLSs, which comprise “several smooth, elongated plates and some tuberculated, occasionally oval ones” ([28], p. 1306), although the specimens were not illustrated [28]. Subsequently illustrated specimens show some tuberculate plates with a rectangular shape (fig 272 in [31]; fig 487 in [39]). These smooth and tuberculate plates have never been described in detail.

Specimen YKLP 13889 has both smooth and tuberculate plates very well preserved (Figs. 1a–b; 8). There are at least three, probably four, smooth plates preserved in this specimen. They are rectangular and can reach a width similar to podomere 5 of the frontal appendage (sp in Fig. 1a–b). At least the two lower smooth plates connect to each other, indicating that they might have been articulated in life. Sometimes, laterally extended folds are preserved, which nearly cross the whole width of the smooth plates (Figs. 1a–b; 8a). In YKLP 13323, two fragments of smooth plates can be recognized, with cross folds preserved (sp in Figs. 5a, c; 6a, d).

Within the enclosed space of the smooth plates of YKLP 13889, there are tuberculate plates of various sizes, sometimes aligned in rows (Figs. 1v; 8a–c). These plates are characterised by the presence of a dense covering of small (<0.5 mm) flattened, rounded-triangular scales on the surface of the plate. The largest tuberculate plates are adjacent and parallel to the smooth plates, which are slightly smaller in size than the tuberculate plates (Figs. 1a–b; 8a, c). The tuberculate plates are horseshoe-shaped, with at least 11 small triangular spines along the outer curved edge pointing towards the smooth plates (Fig. 8c, e; arrows in Fig. 8d). The inner curved edge is smooth, with a prominent spine at both lateral corners which points to the opposite side (stars in Fig. 8d, e). In the two known rows, the size of the other tuberculate plates decreases successively, with their shape varying from triangular to rod-like or cone shaped (Figs. 1a–b; 8a–c). Like the horseshoe-shaped tuberculate plates, these smaller tuberculate plates also bear small tooth-like spines (Fig. 8b–e). In the left column, some plates bear one or two prominent spines (arrows in Fig. 8b). In YKLP 13323, a row of structures is preserved between the smooth plates and GLSs, with the lowest one bearing small spines along its edge (tp in Fig. 5a, c, d). Together with their pointed lateral corners, this series of structures is also interpreted as tuberculate plates, although no scales can be recognized on them. Some curved linear structures are distinct on these plates (Fig. 5a, c, d).

#### Head carapaces

An oval sclerotized structure was illustrated in the original descriptions of *Amplectobelua symbrachiata*, but was mistakenly interpreted as the carapace of a bivalved arthropod [28] or plates possibly attached to the head [27]. This structure was recently re-interpreted as the head shield of *A. symbrachiata*, which bears a marginal rim along the edge [29]. New material illustrated here confirms that *A. symbrachiata* bears an oval head shield. In most cases, it is preserved in high relief, as indicated by the presence of artefactual wrinkles near the margin (Fig. 4b) and the breakage commonly seen in the central region of the head shield (Figs. 1a﻿–b; 3a–b, f; 4a–b; 5a, f; 6a, d also see figs 18e–f in [29]). The marginal rim has a tendency to narrow from the anterior edge along both lateral sides until it disappears at the posterior edge, which is nearly straight (Figs. 3a, b, f; 6a, d; also see figs 18e–f in [29]).

In addition to the oval dorsal head shield, YKLP 13313b has a pair of prominent ovoid structures preserved adjacent to the frontal appendage. They are in very high relief, set in bilateral symmetry, and bear conspicuous concentric lines on the surface (Fig. 3a, f, h). In YKLP 13323, a pair of ovoid structures is also preserved, with at least one of them in high relief (Figs. 5a, c; 6a, d). A similar pair of structures has been described in the holotype of *Amplectobelua symbrachiata*, and were interpreted as eyes [27]. Re-examination of the holotype shows that the supposed ‘eyes’ are in high relief and composed of two layers separated by the mudstone matrix. Concentric lines similar to those seen in YKLP 13313b are also present at least in one layer (Fig. 4a–b). This evidence indicates that the paired high-relief, ovoid structures are not eyes, but are instead a pair of sclerites interpreted here as P-elements (see Discussion below) similar to those seen in *Hurdia victoria* from the Burgess Shale [19, 20]. In YKLP 13313b, an elongated, slightly narrower rod is located between the paired P-elements, which is nearly twice as wide as the long axis of the P-element. The rod also has some degree of relief and is bone-shaped, with its lateral ends expanded slightly (Fig. 3a, f, h). In the middle of the rod, at least two pairs of linear structures are arranged in bilateral symmetry and meet each other at one side (anterior) of the rod, forming a triangular region that separates the rod into two parts (Fig. 3g, h). A similar rod structure is also present between P-elements in the holotype of *A. symbrachiata* (Fig. 4a–b), although it is relatively narrower than the one in YKLP 13313b, with a width only slightly greater than the long axis of the P-element, and with ends that do not expand as in YKLP 13313b. Unfortunately, the middle part of this rod is covered by the proximal part of the frontal appendage, so it is unknown if it bears a middle triangular region. Given its topological relationship with the P-elements, we tentatively interpret it as the rod structure located between the P-elements in YKLP 13313. The difference in relative proportions is most probably due to the orientation of the rod to the rock bedding. Additionally, a sub-triangular structure preserved adjacent to the frontal appendage in YKLP 13314 (Fig. 2a-b) is interpreted as an isolated P-element, as indicated by the wrinkles on its surface.

#### Body flaps

The body flaps of *Amplectobelua symbrachiata* have been documented as similar in number (11 pairs) and morphology to those of *Anomalocaris saron* based on two nearly complete specimens (figs 3, 4 in [28]). We describe here the third known mostly complete specimen (YKLP 13313) with distinct flaps (Figs. 2d; 3a). Their topological arrangement is recovered by combining information from both part and counterpart (Fig. 3f), with right and left flaps distinguished based on their proximity to the right and left appendages, respectively. This numbering may not reflect the real orientation in life, as the specimen is somewhat disarticulated and distorted. All preserved flaps in YKLP 13313 are approximately 3 cm long (Fig. 3f), which conforms to the size of anterior flaps illustrated in the initial description (fig. 3 in [28]). The flaps of YKLP 13313 show transverse lines (using terminology of [20], also referred to as “strengthening rays” in ([21], p. 596), and “veins” in ([28], p. 1306) on the anterior half, which originate from a longitudinal central boundary line that almost crosses the whole length of the flap and extend to the anteriormost margin, forming an angle ranging from less than 25° (distal) to nearly 65° (proximal). The transverse lines are nearly straight in the proximal region of the flap, but start to curve from the middle to the distal part of the flap. Some flaps (fl-l2 and fl-r3 in Fig. 3f) are composed of two layers, with more pronounced transverse lines on the lower surface (Fig. 3a, f). In the posterior basal region of the right fourth flap, two lobate extensions are preserved, with very fine linear structures at least on the anterior one (fl-r4 in Fig. 3a, f; Fig. 3d). In the most proximal part of the right fourth and fifth flaps, the anterior part extends longer than the posterior part, forming a sharply angled region (fl-r4 and fl-r5 in Fig. 3a, f) that might represent the junction between the flap and the trunk.

Other specimens described here preserve partial body flaps. In YKLP 13323 (Figs. 5 and 6), there are three triangular structures branching off from the narrow region overlapped by GLSs, which are of similar size and arranged in a similar orientation (rf in Figs. 5a, c, d; 6a, d, e) to anterior body flaps. Transverse lines can sometimes be recognized on the surface of these flaps, which are parallel to or forming a sharp angle to the anterior edge of the triangular structures (arrows in Figs. 5b and 6b, c). Similar flaps with transverse lines are also preserved in isolation in the same specimen, with two situated close to the three in the assemblage just described and a third adjacent to the frontal appendage and P-element (Fig. 6a, d). The triangular shape and the presence of transverse lines indicates that these are flaps, although their size in YKLP 13323 is relatively smaller than flaps of other *Amplectobelua* specimens (relative to the frontal appendages). Flaps of reduced size are present posterior to the head in several radiodontans, including *Anomalocaris canadensis* [17], and both species of *Lyrarapax* [13, 18]. These triangular structures (of which there are probably three pairs) are interpreted as the reduced transitional flaps of *Am. symbrachiata.*

## Discussion

### The appendicular nature of GLSs

Originally, GLSs were described as part of the mouth apparatus [28] and were interpreted as inner teeth arranged circularly within the mouth of *Amplectobelua symbrachiata* [31]. However, the fact that the GLSs have an extended basal region (Fig. 2a–d, f-g) challenges this interpretation, as including at least three pairs of GLSs in a circlet or oral cone would require the mouth to have a width at least double the length of the GLS. The only known complete specimens of *A. symbrachiata* indicate that the head region is too narrow (relative to the trunk) (fig 3a in [28]; fig 125 in [30]) to accommodate such a wide mouth on the ventral side of the head. In addition, the GLSs are not found arranged in a circle, but are instead regularly arranged in pairs (Figs. 1a–b; 2a–b; 3a, c, f) or in a row (Figs. 2d, f; 3a, f), with the distal spines of each pair being of the same morphotype. The distal spines of the paired GLSs point towards each other (in two of four known pairs, Figs. 2a–b, e; 3a, c, f), and the distal spines of the GLSs aligned in a row point in same direction (one of four known pairs, Fig. 2d, f; 3a, f). Together with the asymmetrical nature of the GLSs (Fig. 7a–f) and their long stem, it is very difficult to allocate these pairs and rows of GLSs into a functional oral cone. Indeed, GLSs in YKLP 13889 are all located outside the spaced enclosed by the smooth plates (Fig. 1a–b), which we interpret as the mouth edge (see the Discussion on the mouth below). All this evidence indicates that GLSs are paired structures that are located outside the mouth. If this is correct, some questions arise, such as where and how the paired GLSs of *A. symbrachiata* are attached.

When preserved articulated with other body parts, GLSs are always found with the frontal appendages, and sometimes also with the supposed mouth plates and the reduced transitional flaps. This indicates that GLSs are located close to the head and likely facilitate feeding. Such an inference is also supported by the striking similarity of GLSs with the gnathobase (especially the gnathobasic mandible) of euarthropods and some of their upper stem-group taxa (see Discussion below), which are all known or inferred to be feeding structures.

Although it is now well acknowledged that the head of radiodontans ends at the posterior boundary of the protocerebral segment, which bears the eyes [5] (see the Background above), a narrow transitional region with reduced flaps is developed between the head and trunk in both Anomalocarididae and Amplectobeluidae, as seen in *Anomalocaris canadensis* [17] and *An. saron* (figs 1–2 in [28]; fig. 123 in [30]) (Anomalocarididae), and *Lyrarapax unguispinus* and *L. trilobus* [13, 18] (Amplectobeluidae). Material illustrated here shows that *Amplectobelua symbrachiata* has at least three pairs of flaps of reduced size (Figs. 5 and 6), and previously published complete specimens of *Am. symbrachiata* show a narrow transitional region between the head (as indicated by the eyes) and the trunk (i.e., the body flaps) (fig 3 in [28]; fig 125 in [30]). Based on these observations, we propose that a transitional region with several reduced segments might be a shared character in at least Anomalocarididae and Amplectobeluidae. In YKLP 13323 (Figs. 5a, c, d; 6a, d, e), three GLSs of the same size and orientation overlap on the most proximal region of three reduced flaps, indicating that one GLS corresponds or attaches to each flap pair or transitional segment. Given the paired nature of GLSs and the inferred limb affinities of flaps [42], this suggests that GLSs are most likely paired structures with an appendicular nature, with each pair of GLSs having its own segmental affinity. The appendicular interpretation of flaps is potentially strengthened by the finding of lobate extensions from the proximal region (Fig. 3a, d, f), these extensions resembling lobate endites.

### Are GLSs true gnathobases?

A gnathobase is a highly developed spinose or setose endite, typically located along the entire margin of the protopodite (or protopodal podomeres, if the protopodite is jointed) [43], used to manipulate and/or move food. Gnathobases are widely known as the basal part of appendages in crown-group Euarthropoda and their upper stem group, but are not necessarily associated with the endopodite and/or exopodite, both of which are limb branches from the protopodite. In most cases, gnathobases are associated with fully developed appendages (biramous or uniramous), but an exception is the mandible in many Mandibulata, the gnathal edge of which is a gnathobasic remnant of the first post-tritocerebral appendage across all subgroups of Mandibulata. In myriapods and hexapods the mandible consistently lacks a palp/telopodite and the gnathal edge represents a coxal gnathobase, as is the case in some crustaceans as well [44, 45]. The striking similarity between the GLS and euarthropod gnathobases, together with their association with reduced transitional flaps, segmental nature, and possible limb affinity, raises the question as to whether these two structures are homologous. Here we compare the morphology of GLSs with a range of panarthropod taxa that bear similar feeding structures, and the jaws of onychophorans.

#### *Comparison with the gnathobases of* Parapeytoia

*Parapeytoia yunnanensis* was originally described as a radiodontan due to an inferred radial oral cone [27]. However, the described biramous appendages with a jointed endopodite, pronounced gnathobasic endites, and highly sclerotized sternites have brought the radiodontan assignment into question, with most cladistic analyses placing it as an upper stem-group euarthropod within Megacheira (e.g. [46]), although its radial oral cone conflicts with that interpretation. With its high quality preservation (Fig. 9b), the gnathobase of *P. yunnanensis* is the best candidate for comparison with the GLSs.

Originally, five weakly defined gnathobasic endites were described along the protopodite (propod of [27]) of each biramous appendage of *Parapeytoia yunnanensis* (Fig. 9b), with each endite bearing three or four distal spines. However, without direct evidence for segmental boundaries, it is open to debate whether each cluster of distal spines represents a separate gnathobase. For example, the gnathobase of *Sidneyia inexpectans* also bears several clusters of spines, with each cluster defined by a larger spine [47]. Thus the original five “gnathobases” of *P. yunnanensis* are interpreted as five clusters of spines. When curved, the distal spines point to the attachment side of the endopodite, which is interpreted as the inner side, and the opposite side as outer. The outermost distal spine of each cluster is prominently larger than the others, with decreasing size towards the inner ones. In the outermost cluster (cluster 5), the outer edge expands laterally. A double layered margin, at least at the base of the largest distal spine of each cluster, can be easily recognized (solid arrows in Fig. 9b), indicating that the distal spines might be situated within sockets of the gnathal edge. Scales are present in the gnathal blade (hollow arrows in Fig. 9b).

The gnathobase of *Parapeytoia yunnanensis* is quite similar to the GLS of *Amplectobelua symbrachiata*, sharing features such as the sockets along the gnathal edge and the scales on the gnathal blades (Fig. 9a, b), but with a different arrangement of distal spines. The long stem of GLSs is also comparable to the expanded protopodite region of *P. yunnanensis*, but in the latter taxon only the protopodite region opposite the gnathobase is expanded (branched) to form an exopodite that was interpreted as having a flap-like shape, while immediately adjacent to the most inner gnathobase, limb podomeres bearing spinose endites form the jointed endopodite. Although GLSs are inferred to be appendicular structures attached on the reduced transitional segments of *A. symbrachiata*, presently there is no evidence for an endopodite (or comparable structure). Thus it is currently premature to homologize GLSs of *A. symbrachiata* with the gnathobase of *P. yunnanensis*.

#### Comparison with the mandible/gnathobase of euarthropods

Gnathobases are present on a variable number of appendages in euarthropods. A gnathobase may be present only on the mandible (as seen in insects and myriapods), or they may be developed on an extended series of appendages (e.g., prosomal limbs II-VI in Xiphosura: Fig. 9d). A gnathobase has also been well documented in many Cambrian Euarthropoda, such as Megacheira, Artiopoda and probably some bivalved arthropods (e.g. *Canadaspis laevigata*) [48], among which the morphology varies owing to differences in feeding habits. In many cases, gnathobases are associated with all known post-antennal appendages in these fossil arthropods, as seen in *Naraoia*, trilobites, and *Leanchoilia* [48]. The strongly dentate inner margin of GLSs of *Amplectobelua symbrachiata* particularly resembles the pars incisivus of many mandibles (Fig. 9e–g). Comparisons can be made, for example, with the groups of teeth in the mandibles of centipedes (Fig. 9e), which function as cutting carnasial teeth [49]. Given that the mandible is specialised as the main mouthpart of the adult head in Mandibulata, has a precise segmental identity (as the appendage of the post-tritocerebral segment), and is accommodated in a chewing chamber in a highly modified head, whereas the GLSs of *A. symbrachiata* are associated with multiple segments, homology with mandibles can go no deeper than considering them both as segmental appendages. Nevertheless, the morphological similarities between mandibles and GLSs allow for functional inferences. Mandibles are highly diverse with respect to function: in addition to cutting and biting they variably also serve to scratch surfaces for food, squeeze or grind food, or hold or pierce prey. As noted above, the development of a series of relatively few strong teeth along the gnathal edge of the GLS is comparable to extant euarthropod mandibles in which cutting is a primary function of the pars incisivus. However, gnathal edges composed of a row of strong teeth can have specialised functions. As an example, some copepods use the dentate gnathal edge of the mandible to crack the resistant tests of diatoms that serve as their principal source of food [50] (Fig. 9f–g).

#### Comparison with the jaws of onychophorans

Onychophora have two pairs of jaw blades within their mouth cavity [51], the gross morphology of which bears comparison to GLSs of *Amplectobelua symbrachiata*. The onychophoran mouth is surrounded by lip papillae that have a radial appearance but their innervation reveals them to be bilaterally symmetrical, with contributions from three segments [52]. The jaws are the appendages of the deutocerebral segment, becoming incorporated into the mouth cavity during embryonic development (fig 1 in [53]). Shared morphological details with the claws of the body appendages reveal that the jaws and claws are serial homologues, demonstrating that the jaw represents the distal part of an appendage, rather than being a “whole limb” [51].

Onychophoran jaws consist of an outer and an inner blade (fig 3 in [51]). The outer blade has a sickle-shaped principal tooth and variably has smaller accessory teeth, whereas the inner blade has a similar principal tooth that may likewise be associated with accessory teeth but also bears a prominent row of denticles (Fig. 9c). These strongly sclerotized jaws, especially the inner blade with its strong teeth, compare with GLSs, the most profound difference being that they are situated within the mouth cavity in Onychophora whereas, as argued above, this does not appear to be possible in *Amplectobelua symbrachiata*. The onychophoran comparison, like that made above for euarthropod mandibles, is possibly mostly informative for inferring the likely function of GLSs.

#### Homology with euarthropod gnathobases uncertain

In summary, GLSs resemble gnathobasic mouthparts of euarthropods. The interpretation of the GLSs as appendicular feeding structures of the transitional region at the posterior of the head is in some ways paralleled by examples in extant euarthropods. For example, the most jaw-like gnathobases in Xiphosura are those of prosomal limbs furthest posterior, most distant from the mouth. The robustly-armoured gnathobase of leg VI in *Limulus* plays a role in cracking stiffer food that is then shredded by the gnathobases of the preceding prosomal appendages (legs III-V) [49]. The differences in the GLSs of *Amplectobelua symbrachiata* may likewise reflect differences in function as food is sliced and otherwise processed on its way towards the mouth. Whether GLSs are true gnathobases depends in part on how they are attached to the reduced transitional segments, i.e. the topological relationship with the (reduced) flaps that likely have limb affinities [42]. Additionally, given the lack of external indications of subdivisions in the GLSs, it cannot definitively be stated that they are indeed outgrowths of a protopodite or other such proximal region of an arthropodized appendage. The similarity to the dentate jaws of Onychophora leaves open the possibility that they could instead represent the distal part of an appendage, as may also be suggested by the elongated nature of the GLSs. We thus informally refer to them as gnathobase-like structures and leave their affinity with gnathobases open to debate.

### The mouthparts of *Amplectobelua symbrachiata* are not of the *Peytoia*-type

The name Radiodonta was derived from the prominent and highly regular 32-plate oral cones of the first taxa described from whole body specimens, *Anomalocaris canadensis* and *Peytoia nathorsti* from the Burgess Shale [21, 23, 54]. These oral cones consist of four large semi-rectangular plates arranged perpendicular to one another, with seven smaller plates between them, surrounding a square or rectangular central opening. It was thought that this type of oral cone was consistently present in the then known radiodontan taxa [21, 23], but recent research has shown that radiodontan mouthparts are actually highly variable [22]. *Hurdia* has the typical 32-plate oral cone, but with additional rows of spinose plates within the central opening [19, 20]. *Anomalocaris* (including *A. canadensis* from the Burgess Shale [17, 22], *Anomalocaris* sp. from the Emu Bay Shale [55], and *A. saron* from the Chengjiang Biota [27, 28]) has been shown to have a flexible oral cone consisting of three large plates with variable numbers of smaller plates between them, all of which bear radially arranged furrows on their outer margins. The Chengjiang taxon *Lyrarapax* does not have an oral cone with plates, but instead has mouthparts consisting of concentric folds [13, 18]. As such, the only taxon with the “classic” 32-plate oral cone (without inner spinose plates) is *Peytoia nathorsti* of the Burgess Shale [22].

The material described here for *Amplectobelua symbrachiata* shows yet another morphology for the mouthparts of radiodontans. Although a fully articulated oral structure is not preserved, the disarticulated specimens described here suggest that it consists of an outer arrangement of smooth plates surrounding inner rows of tuberculate plates (Fig. 1a–b). In basic structure, this is most similar to the oral cone of *Hurdia victoria* [19, 20] which also has smooth outer plates and toothed inner plates, but the details of the arrangement, shape and orientation of the components differ considerably between these two taxa. While the 32 outer plates of *H. victoria* are sub-rectangular and taper towards the inner opening, the smooth plates of *A. symbrachiata* are fewer in number (only four) and are elongate and arcuate in shape. The folds observed in the outer plates of *A. symbrachiata* are similar to the longitudinal wrinkles seen in the outer margins of the oral cone plates in *H. victoria*, which are interpreted as having been caused by the flattening of the three-dimensional dome shape of the oral cone [20]. Furrows in outer plates are also seen in *Anomalocaris* oral cones, but these are anatomical characteristics based on their radial arrangement, regular spacing and deeper extension into the plate [22], as compared to the taphonomic folds seen in *A. symbrachiata*. The tuberculate plates of *A. symbrachiata* are comparable to the inner spinose plates of *H. victoria*, with both being arcuate plates bearing around 9–11 small triangular spines on the inner margin, with at least three or four plates arranged in overlapping rows. In *H. victoria* there are four sets of multiple rows of spinose plates, arranged perpendicular to one another and lining the four sides of the central opening, whereas in *A. symbrachiata* the arrangement is not known for certain, but only two, or possibly three, rows are present (Fig. 1a–b). Also, the prominent scales of the tuberculate plates of *A. symbrachiata* are completely lacking in *H. victoria* [20], although they bear some similarities to the tubercles visible on the oral cone plates of *Anomalocaris* specimens from the Burgess Shale [22], the Emu Bay Shale [55], and Chengjiang [27, 28].

The mouthparts of *Amplectobelua symbrachiata* are unique in structure but display several characteristics recognisable in other radiodontan taxa, combining an overall arrangement similar to *Hurdia victoria* with ornamentation similar to *Anomalocaris*. As is seen in other radiodontans, these structures are associated with features of the head, specifically the frontal appendages and carapace elements. The gut in the only articulated body specimen of *Amplectobelua* ends immediately behind the frontal appendage, giving an approximate location for the mouthparts (fig 3a in [28]) despite the absence of smooth and tuberculate plates in this specimen. Radiodontan mouthparts are generally rare within the Chengjiang Biota, and *A. symbrachiata* is no exception, with only a single detailed specimen with mouthparts described to date (Figs. 1a–b; 8), and fragments of tuberculate plates visible in one previously published specimen (fig 3b in [28]). The rarity of radiodontan mouthparts preserved in the Chengjiang Biota in general, as compared to the Burgess Shale where tens of isolated oral cones are well preserved [21], may be the result of a taphonomic filter specific to these localities.

### Head carapaces in radiodontans

*Amplectobelua* is shown here for the first time to have a head carapace structure consisting of three main elements, an oval central head shield and a pair of lateral P-elements in the form of ovoid carapaces with narrow central rods. The oval central head shield of *Amplectobelua* [29] is comparable in shape, size and structure to the dorsal head shield of *Anomalocaris canadensis* from the Burgess Shale [17] and *Anomalocaris saron* from the Chengjiang biota [28]. The central head shield for all these taxa is oval, less than half the length of the frontal appendage in size, and bears a marginal rim. The domed nature of the central head shield is indicated by the high relief preservation in the Chengjiang material, with some lack of preservation of carapace in the central region where relief was highest (Figs. 1; 3a, b, f; 4; 6a, d; also see fig 18e, f in [29]), or by arcuate marginal wrinkling in the Burgess Shale material [17].

The interpretation of the ovoid sclerites of *Amplectobelua* as P-elements is sustained by their shape, paired bilateral symmetry, and topological relationship with the head shield, in comparison to the well-known lateral carapace elements of *Hurdia* from the Burgess Shale [19, 20] and *Aegirocassis* from Fezouata [42, 56]. We question the radiodontan affinity of the isolated lateral elements described in [29] (P-, Z-, and A-elements in fig 1h–j of [29]), and limit our discussion to radiodontan lateral elements found in articulated body specimens. In the most completely articulated *Amplectobelua* specimen from Chengjiang (Fig. 3h), the arrangement of the two P-elements is nearly identical to paired P-elements of *Hurdia victoria* from the Burgess Shale that are preserved joined and flat (fig 9c, h in [20]). In this arrangement, both the *Amplectobelua* and *Hurdia* P-elements are bilaterally symmetrical with their narrower regions oriented centrally and the wider regions directed outwards. However, the structures differ in the location of their articulations. In *Hurdia*, the two P-elements of the pair articulate at their narrow protrusions, with each P-element consisting of a single, unbroken carapace where the narrow anterior protrusion widens out into a roughly rectangular central region (fig 9a–c, g, h in [20]), which bears a posterior notch in some (fig. a-b, g, h in [20]) but not all (figs 1f, g; 5a; 11c in [20]) specimens. The two P-elements of the pair are separated from each other at a break in the anterior notch region. This structure differs from *Amplectobelua*, in which the central region is roughly oval and is a completely separate structure from the narrow rod portion, which is equivalent in location and orientation to the anterior protrusion of the *Hurdia* material. In *Amplectobelua*, the narrow rod region is a single structure (equivalent to the anterior protrusions of two separate *Hurdia* P-elements), and the rod structure is articulated with a separate oval structure on either side. The break between the central oval region and the rod structure is consistent between specimens (Figs. 3f, h; 4a–b). The triangular region located centrally in the rod structure in *Amplectobelua* (Fig. 3g) is exactly equivalent in position to where the margins of the anterior protrusions of the two separate P-elements meet in *Hurdia*. The differing articulation between *Hurdia* and *Amplectobelua* P-element components does not detract from their remarkably similar outlines when fully articulated, but produces isolated elements of very different outlines when disarticulated (for *Hurdia*, isolated elements are rectangular with an attached anterior protrusion, but for *Amplectobelua* isolated elements consist of either rod-shaped or ovoid elements). The articulation arrangement of the P-element of *Amplectobelua* may be more similar to the lateral element of *Aegirocassis* from Fezouata, which consists of a large, domed ovoid carapace with an offset and upturned anterior projection that may articulate separately [42].

Compositionally, the P-elements of *Amplectobelua* are similar to *Hurdia* H-elements [20] in that they both consist of two layers of cuticle separated by sediment. The P-elements of *Hurdia* [20] and *Aegirocassis* [42] do not exhibit this double-layer structure (in contrast to the claims of table 3 in [29]). *Hurdia* P-elements also uniquely have a polygonal reticulate pattern covering the surface of the carapace, preserved as reflective films or narrow ridges or valleys [20], but this ornamentation was not identified in the material of *Amplectobelua* described here. In general, the lateral P-elements of *Amplectobelua*, *Hurdia*, and *Aegirocassis* share a similarity in overall symmetry, shape, and location on the body, but vary in ornamentation and ultrastructure between taxa.

A previously described specimen of a radiodontan assemblage from Chengjiang (NIGPAS 162524; fig 1j, 12 in [29]) is the only other known specimen from these localities to have a pair of P-elements (referred to as aliform sclerite elements (A-elements) in [29]). The carapaces of this specimen are more similar in overall arrangement and outline to *Hurdia victoria* from the Burgess Shale than they are to *Amplectobelua symbrachiata*, with each P-element of the pair consisting of a single, unbroken carapace with a poorly defined narrow anterior protrusion and a wider central region (fig 12a–d in [29]). A triangular structure (JS in fig 12d of [29],) separates the two anterior protrusions, similar to the triangular structure of the P-element rods of *A. symbrachiata*, and the carapaces of NIGPAS 162524 also exhibit a double-layer structure. Five-toothed sclerites found associated with the P-elements are similar to the GLSs described here for *A. symbrachiata*, but have a different spine arrangement that more smoothly increases in size and convexity, and are arranged with three elements in a row (TO5 and TO5? in fig 12c of [29]). Tuberculate plates are also present (fig 12c, h in [29]), as well as a robust plate with three teeth and prominent nodes (fig 12l in [29]) and large setal blades [17] that consist of lanceolate blades attached together along one edge (fig 12i in [29]). This combination of structures suggests a radiodontan affinity for this specimen, but we do not consider it to be *A. symbrachiata* because of the lack of frontal appendages, the different morphology of the P-elements and GLSs, and the presence of structures not usually preserved with *A. symbrachiata* (i.e. a robust plate with nodes and setal blades).

Head carapaces in radiodontans have been identified in nearly every taxon known from disarticulated or articulated body specimens (more than just isolated appendages), and the morphology of these structures was thought to be distinct between the two major radiodontan clades, Hurdiidae and Anomalocarididae + Amplectobeluidae [13, 33, 42]. Hurdiidae was thought to be characterised by the presence of a three-part frontal carapace consisting of a central element and two mirror-image lateral elements, while Anomalocarididae + Amplectobeluidae was characterised by the presence of a single, oval dorsal head shield. The new specimens of *Amplectobelua* described here show that the three-part frontal carapace is not restricted to *Hurdia* and other similar taxa, but can also be found within Amplectobeluidae. The presence of the typical oval dorsal head shield of Anomalocarididae + Amplectobeluidae is maintained, but we show here the additional lateral carapace elements. As is seen in other radiodontan taxa, the lateral P-elements and the dorsal head shield of *Amplectobelua* are anterior cephalic structures, reinforcing their interpretation as derived protocerebral structures that are not homologous to bivalved carapaces or other carapace features in the more crownwards Deuteropoda [13, 33, 42].

The morphology of head carapaces has contributed to discussions on the ecological interpretation of different radiodontan taxa [29]. Based on functional morphology of their appendages, it has been suggested that taxa such as *Anomalocaris* and *Amplectobelua* were active predators [29], and that their dorsal cephalic carapace must be small in order to maintain a wide range of movement for the frontal appendages. In contrast, taxa such as *Hurdia* and *Aegirocassis* have appendages with a limited range of motion that indicate a more generalised sweep-feeding [33] or filter-feeding [42] habit, and the large frontal carapace might have helped to trap prey items and funnel them towards the mouth [20]. This may explain why the lateral elements described here for *Amplectobelua* are much smaller than those of *Hurdia* or *Aegirocassis*, with the P-elements of *Amplectobelua* being no longer than the length of the frontal appendage whereas the P-elements of *Hurdia* and *Aegirocassis* are 5–6 times longer than the frontal appendage at least [20, 42]. The much smaller lateral elements in *Amplectobelua* likely did not limit the range of motion of the frontal appendages during active predation, but may have provided additional protection against damage in the cephalic region.

### Phylogenetic significance

Despite being widely accepted as arthropods, the precise phylogenetic position of radiodontans within the total-group Panarthropoda is less secure. The prevailing hypothesis is that they are a branch in the stem group of Euarthropoda, intermediate between the gilled lobopodians and the upper-stem euarthropods [57–60], a result that has been retrieved by almost all cladistic analyses based on explicitly coded morphological matrices (e.g. [46]). Morphologically, this hypothesis is supported by several key characters that bridge morphological gaps between the gilled lobopodians and the upper-stem euarthropods [61], e.g., the frontal appendages are homologous with the primary antenna of lobopodians (including onychophorans) [8, 13, 57, 59, 60], the flaps are comparable with those of gilled lobopodians [42, 57–60], the digestive glands are similar to those of both gilled lobopodians and euarthropods [25], and the compound eyes are more similar to those of euarthropods [62, 63]. In this scenario, the head structure of *Amplectobelua symbrachiata* additionally provides a set of key characters of the head region that link the lower and the upper-stem euarthropods. The reduced transitional segments that bear reduced flaps and appendicular GLSs in *A. symbrachiata* indicate that integration of additional body segments into the head region might have occurred before the establishment of the three-segmented head pattern of Deuteropoda. The mouth parts of *A. symbrachiata*, which are different from the typical radial oral cones (*Anomalocaris*, *Hurdia*, *Peytoia* et al.) or the mouth of *Lyrarapax*, further demonstrate the morphological diversity of the mouth of radiodontans. Such a diversity of mouth morphology opens a window to speculate how the mouth apparatus evolved from the lower stem to the upper stem group of Euarthropoda.

The evolutionary significance of our findings can be interpreted differently in an alternative scenario in which radiodontans are crown-group euarthropods, and more precisely are stem-group chelicerates. To date, this hypothesis has relied only upon morphological similarity of the frontal appendages of radiodontans with those of megacherians and chelicerates [14, 15]. In this scenario, the appendicular GLSs of *Amplectobelua symbrachiata* would be comparable with the gnathobases of megacherians and chelicerates, while the head carapaces of *A. symbrachiata* can be homologized with a sclerotised tergal exoskeleton, a euarthropod apomorphy. However, given the lack of convincing body appendages in radiodontans, and assuming this absence to be plesiomorphic in Radiodonta, this hypothesis would require that jointed body appendages evolved independently in Chelicerata and Mandibulata.

## Conclusions

Documentation of new specimens of the most common Chengjiang radiodontan, *Amplectobelua symbrachiata*, reveals many new details of the morphology of this species, until now largely known from its frontal appendages. These elucidate not only the morphology of this emblematic species but provide novel character states and combinations of characters for Radiodonta as a whole. Some of these contribute to considerations of phylogenetic relationships within the group, whereas others need be considered in broader questions of homologies and the debate over the stem- or crown- group position of Radiodonta within Euarthropoda. A dorsal cephalic shield that had only recently been attributed to this species is shown to be supplemented by paired cephalic sclerites that are homologised with P-elements of hurdiids (Fig. 10). As such, the cephalic sclerites share characters with Anomalocarididae (ovoid dorsal head shield) and Hurdiidae (paired P-elements). The mouth apparatus, composed of smooth and tuberculate plates, deviates from the “*Peytoia*” oral cone of most other radiodontans, although comparison with *Hurdia* in particular allows some correspondences to be proposed. *A. symbrachiata* possessed a transitional region at the posterior of the head, composed of three segments with reduced flaps. Gnathobase-like structures that were previously interpreted as parts of the oral cone are instead associated with the reduced transitional segments. The gnathobase-like structures can be identified as two different morphotypes, comprising three segmental pairs that are inferred to play a role in cutting and shredding food in its passage to the mouth opening (Fig. 10). The correspondences in the morphology of the gnathobase-like structures of this radiodontan and gnathobasic appendages of euarthropods corroborates the appendicular nature of these structures. Their association with the reduced transitional flaps opens up the question as to precisely what part of an appendage they represent. The discovery of more completely articulated material may shed light on this question, and solve the puzzle as to whether the gnathobase-like structures bite with the proximal or distal part of the appendage. Our study reveals that *Amplectobelua* had a more elaborate set of cephalic sclerites than was previously known and had gnathal appendages associated with a reduced transitional region that was effectively cephalised, as part of the functional head (Fig. 10).

## Methods

New fossil material was prepared with fine needles under a Nikon SMZ 800N stereomicroscope with incident light to reveal the morphology covered by the mudrock matrix. All fossil material and the gnathobase of *Limulus polyphemus* were investigated with the same stereomicroscope under both incident light and/or polarized light, then photographed under cross polarized light with a Canon 650D camera mounted with a Canon EF-S 60 mm macro lens or a Canon MP-E 65 mm (1-5X) macro lens. Camera lucida drawings were made with a Meiji Techno RZ stereo microscope and traced in Adobe Illustrator CC 2014, with evidence from different images sometimes integrated. SEM images of *Ethmostigmus rubripes* were taken with a Leo 435VP SEM with a Robinson backscatter collector. Brightness/contrast and the tone of all images were refined by optimizing the levels in Adobe Photoshop CC 2014. The figures were prepared with Adobe Photoshop CC 2014.

### Terminology

Throughout the manuscript, taxonomic terminology strictly follows that of Ortega-Hernández, 2016 [6]. Panarthropoda refers to a clade including Euarthropoda, Onychophora and Tardigrada [2]. Euarthropoda sensu Lankester, 1904 [32] consists of the clade including the most recent common ancestor of extant chelicerates, myriapods, and pancrustaceans and all of its descendents, to the exclusion of Onychophora and Tardigrada [6, 64]. Lower stem-Euarthropoda consists of organisms with lobopodian-type body construction (lobopodians, gilled lobopodians), the enigmatic taxa *Opabinia* and *Schinderhannes*, and Radiodonta, while upper stem-Euarthropoda includes fuxianhuiids, bivalved stem-group euarthropods, and megacheirans. Deuteropoda is a scion comprising Euarthropoda (the crown group) and upper stem-Euarthropoda, and is defined anatomically by the presence of a “structurally differentiated deutocerebral first appendage pair, reduced protocerebral appendages integrated into the labrum/hypostome complex, and a multisegmented head” ([6], p. 269). Radiodonta refers to a clade of fossils with a large pair of jointed frontal appendages, concentric circumoral structures (plates or folds/furrows), eyes on stalks, and a trunk consisting of a series of paired swim flaps. Radiodonta includes the Family Anomalocarididae (*Anomalocaris* and closely related taxa), Family Hurdidae (*Hurdia, Peytoia,* and closely related taxa) and Family Amplectobeluadae (*Amplectobelua* and closely related taxa). As such, the frequently used term “anomalocaridids” refers to only a subset of radiodontans.

Anatomical terminology follows recent radiodontan publications, namely [17, 18, 42]. The term “head carapace” or “cephalic carapace” refers to all sclerites located in the head region, including both the P-elements [19] (“lateral elements” of [42]) and the central head shield (“H-element” of [19]; “central element” of [42]). As is used by [65], the term “podomere” is used to refer to the individual articles of the jointed/arthropodized appendages of arthropod fossils, including the frontal appendage of radiodontans. Radiodonta swim flaps bear “transverse lines” [20], also referred to as “strengthening rays” ([21], p. 596) or “veins” ([28], p. 1306) in previous publications.

### Abbreviations in figures

as, auxiliary spine; ds, dorsal (outer) spine; en, endite; fl, flap; glsA/B, gnathobase-like structures (GLS), with two morphotypes (morph-A and morph-B); gs, spines at distal end of GLS; hs, head shield; is, small spines at inner side of GLS; pd, podomere; pe, P-element; rf, reduced flaps on the transitional segments; rp, rod-shaped scleritization between P-elements; rs, reduced transitional segments; sc, scale-like ornamentation on stem of GLS; sp, smooth plates; tp, tuberculate plates; ts, top apical spine.
